# Functional and structural investigation of a broadly neutralizing SARS-CoV-2 antibody

**DOI:** 10.1172/jci.insight.179726

**Published:** 2024-05-22

**Authors:** Yi-Hsuan Chang, Min-Feng Hsu, Wei-Nan Chen, Min-Hao Wu, Wye-Lup Kong, Mei-Yeh Jade Lu, Chih-Heng Huang, Fang-Ju Chang, Lan-Yi Chang, Ho-Yang Tsai, Chao-Ping Tung, Jou-Hui Yu, Yali Kuo, Yu-Chi Chou, Li-Yang Bai, Yuan-Chih Chang, An-Yu Chen, Cheng-Cheung Chen, Yi-Hua Chen, Chun-Che Liao, Chih-Shin Chang, Jian-Jong Liang, Yi-Ling Lin, Takashi Angata, Shang-Te Danny Hsu, Kuo-I Lin

**Affiliations:** 1 Genomics Research Center, Academia Sinica, Taipei, Taiwan.; 2 Institute of Biochemical Sciences, National Taiwan University, Taipei, Taiwan.; 3 Institute of Biological Chemistry and; 4 Biodiversity Research Center, Academia Sinica, Taipei, Taiwan.; 5 Institute of Preventive Medicine,; 6 Graduate Institute of Medical Sciences, and; 7 Department of Microbiology and Immunology, National Defense Medical Center, Taipei, Taiwan.; 8 Biomedical Translation Research Center (BioTReC),; 9 Academia Sinica Cryo-EM Center, and; 10 Institute of Biomedical Sciences, Academia Sinica, Taipei, Taiwan.; 11 International Institute for Sustainability with Knotted Chiral Meta Matter (WPI-SKC M^2^) Hiroshima University, Hiroshima, Japan.

**Keywords:** COVID-19, Infectious disease, Adaptive immunity, Immunoglobulins, Structural biology

## Abstract

Since its emergence, SARS-CoV-2 has been continuously evolving, hampering the effectiveness of current vaccines against COVID-19. mAbs can be used to treat patients at risk of severe COVID-19. Thus, the development of broadly protective mAbs and an understanding of the underlying protective mechanisms are of great importance. Here, we isolated mAbs from donors with breakthrough infection with Omicron subvariants using a single–B cell screening platform. We identified a mAb, O5C2, which possesses broad-spectrum neutralization and antibody-dependent cell-mediated cytotoxic activities against SARS-CoV-2 variants, including EG.5.1. Single-particle analysis by cryo-electron microscopy revealed that O5C2 targeted an unusually large epitope within the receptor-binding domain of spike protein that overlapped with the angiotensin-converting enzyme 2 binding interface. Furthermore, O5C2 effectively protected against BA.5 Omicron infection in vivo by mediating changes in transcriptomes enriched in genes involved in apoptosis and interferon responses. Our findings provide insights into the development of pan-protective mAbs against SARS-CoV-2.

## Abstract 2

A near pan-protective SARS-CoV-2 mAb binds the Omicron RBD with an exceptionally large interface that encompasses the complete ACE2-binding site and surrounding regions, as compared with known class I antibodies.

## Introduction

SARS-CoV-2 belongs to the positive-sense single-stranded RNA beta-coronavirus group, whose essential structural components include the spike (S) protein, membrane (M) protein, envelope (E) protein, and nucleocapsid (N) protein. The entry of the virus into host cells is facilitated by the S protein, which forms transmembrane homotrimers protruding from the viral surface (1). The S protein is a crucial target for antiviral strategies because of its role in viral entry (2). It consists of 2 functional subunits: S1 and S2. The S1 subunit includes the N-terminal domain (NTD) and the receptor-binding domain (RBD). The primary function of the S1 subunit is to attach to the key receptor, angiotensin-converting enzyme 2 (ACE2), on host cells. The S2 subunit contains various domains, including the fusion peptide (FP), heptad repeat 1 (HR1), central helix, connector domain, heptad repeat 2 (HR2), transmembrane domain (TM), and cytoplasmic tail. The S2 subunit mediates membrane fusion (3). During the COVID-19 pandemic, SARS-CoV-2 continuously evolved mutations (4). The Omicron variant of SARS-CoV-2, carrying 32 amino acid mutations in the S protein (B.1.1.529), was first detected in South Africa and Botswana in November 2021 and then rapidly spread worldwide (5). Thus far, Omicron has evolved a remarkable number of sublineages and is currently divided into 5 major lineages: BA.1, BA.2, BA.3, BA.4, and BA.5 (6). Several descendant lineages of BA.2 then emerged, including XBB.1.5, XBB.1.16, EG.5, BA.2.86, and the recent JN.1, which were listed by WHO as variants of interest because of its rapid growth rate and immune evasion (7).

Humoral immunity plays an important role in the protection against SARS-CoV-2 infection (8). S protein binding antibodies elicited during infection enter the blood circulation and peripheral tissues to deplete the virus through neutralization (9, 10). Upon SARS-CoV-2 infection, preexisting cross-reactive memory B cells targeting both seasonal beta-coronaviruses and SARS-CoV-2 S proteins undergo extrafollicular activation to generate short-lived plasma cells for early protection (11). Simultaneously, naive B cells specific to SARS-CoV-2 S protein are activated, with the help of T follicular helper cells, then are recruited into the germinal centers for clonal expansion, antibody class-switching recombination, and somatic hypermutation for affinity maturation, resulting in the generation of long-lived plasma cells and memory B cells for long-term protection (10–12). Progressive accumulation of mutations in antibodies specifically targeting RBD in convalescence did not abrogate the overall neutralization capability against SARS-CoV-2 (11). Prolonged or repeated SARS-CoV-2 antigen exposure promotes somatic hypermutation and affinity maturation and results in the production of cross-neutralizing antibodies (13, 14). In addition to neutralizing activity, antibodies possess Fc domain–dependent effector functions, including antibody-dependent cellular cytotoxicity (ADCC) and antibody-dependent cellular phagocytosis (ADCP), induced by their interaction with Fc receptor (FcR) expressed on NK cells and macrophages/monocytes, respectively. The Fc domain recruits complement components for antibody-dependent complement deposition (ADCD) to lyse infected cells (15). Fc–FcR engagement between neutralizing antibodies and immune cells is necessary for full protection against SARS-CoV-2 infection, demonstrating the significance of both neutralization and Fc domain–dependent effector functions (16).

SARS-CoV-2–specific mAbs are one of the most effective options for treating individuals with severe COVID-19 or those unable to receive vaccines (17). Several mAbs have been developed for use since the outbreak of SARS-CoV-2, including sotrovimab, bebtelovimab, the cocktail Ronapreve (casirivimab + imdevimab), a combination of cilgavimab and tixagevimab, and a combination of bamlanivimab and etesevimab; however, newly emerging Omicron subvariants have been shown to be resistant to the protective effects of these mAbs as a result of S protein mutations and immune evasion (18). Several mAbs showing neutralizing activity against Omicron subvariants of BA.1, BA.2, and BA.4 have been identified, but little is known about the effectiveness of mAbs against XBB.1.16 or other recently evolved Omicron sublineages, including EG.5.1. Furthermore, the molecular mechanisms by which broadly protective mAbs function in vivo to confer protection against SARS-CoV-2 infection remain elusive.

In this study, we isolated a mAb, O5C2, recognizing the RBD region of S protein, that showed broad-spectrum protection against various Omicron sublineages in vitro and in vivo, as well as protection against all tested SARS-CoV-2 variants. To delineate the structural basis of the broad-spectrum neutralization activity of O5C2, we carried out cryo-electron microscopy (cryo-EM) single-particle analysis to determine the structure of the S protein of the BQ.1 variant in complex with ACE2 or O5C2. The atomic structural models revealed that O5C2 targets a large epitope within the RBD that overlaps with the ACE2 binding interface. Furthermore, RNA-Seq analysis using lung tissue from O5C2-treated mice showed the substantial activity of O5C2 in vivo, involving upregulation of stress responses, apoptotic cell death, and leukocyte migration and downregulation of defense responses to the virus. Our findings reveal the molecular mechanisms of action and the effects of protective mAbs against Omicron sublineages.

## Results

### Identification of mAbs from donors who recovered from SARS-CoV-2 Omicron breakthrough infection.

**Figure 1:**
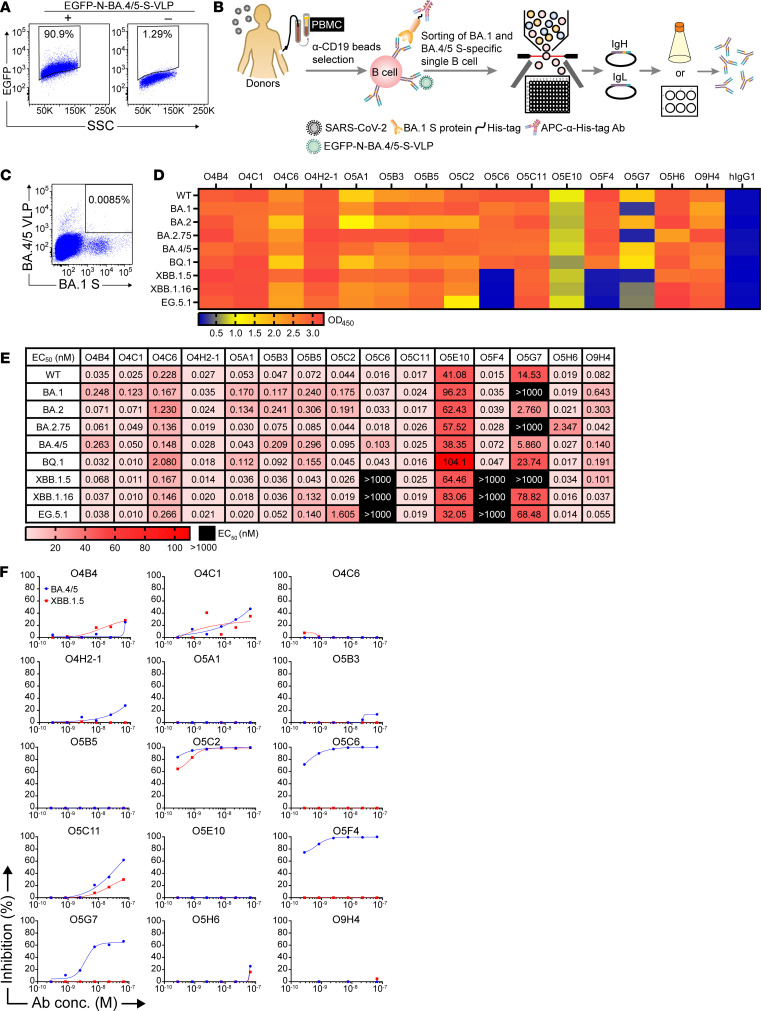
Isolation of mAbs recognizing BA.4/5 and BA.1 SARS-CoV-2 S proteins from PBMCs from recovered individuals. (**A**) FACS showing the binding of EGFP-N-BA.4/5-S-VLP to ACE2-HEK293T cells. (**B**) The diagram depicts the experimental design. PBMCs purified by CD19 magnetic beads from convalescent donors were stained with the EGFP-N-BA.4/5-S-VLP and the His-tagged BA.1-S trimer proteins, followed by single-cell sorting with a cell sorter. The variable regions of immunoglobulin heavy (IgH) and light (IgL) chains were cloned, and the resulting paired plasmids were coexpressed in Expi293F cells to produce mAbs. (**C**) Representative FACS results showing B cells corecognizing EGFP-N-BA.4/5-S-VLP and the trimeric BA.1-S ectodomain. (**D**) Data from ELISA showing the heatmap of the OD readings of various mAbs at a dose of 2.5 μg/mL binding to S protein of the indicated variants of SARS-CoV-2. (**E**) The EC_50_ values (nM) of mAbs from **D** are indicated. (**F**) Screening of the ability of various doses of isolated mAbs in **D** and **E** to neutralize the BA.4/5 or XBB.1.5 pseudotyped virus infection of hACE2-293T cells. The EC_50_ values in **E** were calculated by fitting a curve using a 4-parameter dose-response nonlinear regression analysis.

First, we attempted to identify mAbs that broadly recognize and neutralize the SARS-CoV-2 Omicron sublineages (19, 20). Studies have reported that vaccination with S protein–expressing virus-like particles (VLPs) with a non–SARS-CoV-2 viral backbone elicits higher neutralizing potency than immunization with soluble S protein alone, indicating that the viral surface–anchored S protein may confer advantages in terms of positioning of the overall structure and antigen presentation to immune cells (21, 22). Previous studies also showed that SARS-CoV-2 VLPs could be visualized by expressing GFP-labeled N protein, which can be incorporated during viral assembly in the viral life cycle (23). Therefore, we used the strategy of generating EGFP-conjugated N protein–packaged SARS-CoV-2 VLPs to isolate the S protein–recognizing B cells through FACS because this approach preserves more of the morphology of the authentic virus and the native conformation of S protein (23, 24). EGFP-tagged N and BA.4/5 S protein–expressing (with identical amino acid sequences for the S protein of the BA.4 and BA.5 variants) SARS-CoV-2 VLPs (EGFP-N-BA.4/5-S-VLP) were generated by transfection of Expi293F cells with 4 plasmids expressing BA.4/5 S, E, M, and EGFP-tagged N proteins, respectively (Supplemental Figure 1A; supplemental material available online with this article; https://doi.org/10.1172/jci.insight.179726DS1). The expression of each SARS-CoV-2 structural protein was verified in the purified EGFP-N-BA.4/5-S-VLP (Supplemental Figure 1B). The binding of EGFP-N-BA.4/5-S-VLP to the surface of human ACE2–expressing HEK293T (hACE2-293T) cells was also validated by FACS analysis (Figure 1A) and immunofluorescence imaging (Supplemental Figure 1C).

This EGFP-N-BA.4/5-S-VLP was used in combination with His-tagged recombinant BA.1 S protein to sort peripheral B cells simultaneously recognizing BA.4/5 and BA.1 S proteins (Figure 1B). BA.1 is classified in a different viral clade of SARS-CoV-2 compared with BA.4 and BA.5, which are derived from BA.2, as a result of several unshared mutations in the genome, including mutations in the *S* gene (Supplemental Figure 2) (6). Therefore, mAbs isolated by this approach, together with subsequent single–B cell screening, may be more likely to exhibit broad recognition of the S protein of Omicron subvariants. We selected 3 out of the 8 donors who had recovered from SARS-CoV-2 infection (Supplemental Figure 3A) from whom blood specimens had been collected during September 2022 in Taiwan, a time point when Omicron subvariants were prevalent. The plasma from the 3 selected donors showed more potent neutralization of BA.4/5 pseudotyped virus (Supplemental Figure 3B). Accordingly, we were able to isolate B cells binding to both EGFP-N-BA.4/5-S-VLP and BA.1 S protein though at low frequency (Supplemental Figure 3C and Figure 1C).

Sorted single B cells were further subjected to PCR and cloning of IgH and IgL, followed by mini-scale mAb expression (Figure 1B). The mAbs obtained from mini-scale culture were screened for their binding to Omicron S variants. HEK293T cells stably expressing S proteins (S-293T) from SARS-CoV-2 variants were generated for screening (Supplemental Figure 4). A total of 114 mAbs were generated, and their binding to wild-type (WT, WH01), Delta, BA.1, and BA.4/5 S-293T cells was verified by FACS analysis (Supplemental Table 1). Among the 114 mAbs, 35 (30.7%, >2% binding) bound to S-293T cells expressing any one of the variants tested. On the basis of our FACS screening results, 15 (15/35, 42.9%) of the mAbs showed broad binding ability to the tested S variants and high binding ability to BA.1 or BA.4/5 S protein (>80% binding) (Supplemental Table 1). These 15 mAbs were serially diluted and subjected to ELISA to examine their binding to the S proteins of SARS-CoV-2 variants, including WT, BA.1, BA.2, BA.2.75, BA.4/5, BQ.1, XBB.1.5, XBB.1.16, and EG.5.1 (Figure 1D). The EC_50_ of each mAb to S protein variants was calculated (Figure 1E). Most of our isolated mAbs recognized all S protein variants tested with an EC_50_ below the nanomolar concentration, around the picomolar (pM) level, with the exception of O5C6, O5F4, and O5G7 because of their impeded binding to XBB.1.5, XBB.1.16, and EG.5.1 S proteins (Figure 1, D and E). Next, we used a pseudotyped virus neutralization assay to identify O5C2, O5C6, O5F4, and O5G7 mAbs with potent neutralizing activity against BA.4/5 (Figure 1F). XBB.1.5 S protein harbors a greater number of mutations in the receptor-binding motif (RBM) than the BA.4/5 S protein (20). Only O5C2 was capable of further neutralizing XBB.1.5 pseudotyped virus (Figure 1F). Thus, we used BA.4/5 SARS-CoV-2 VLP harboring EGFP-tagged N protein and BA.1 S protein to isolate several mAbs recognizing the S protein of multiple (sub)variants of SARS-CoV-2. One mAb, O5C2, showed neutralization activity against XBB.1.5 pseudotyped virus infection.

### O5C2 broadly recognizes the S protein of Omicron subvariants.

The neutralizing antibody titers against Omicron subvariants from unvaccinated individuals infected with non-Omicron SARS-CoV-2 variants, uninfected individuals receiving the first generation of SARS-CoV-2 vaccines, and vaccinated individuals with Omicron breakthrough infection have been shown to be lower than those against the D614G variant (25). Indeed, assessment of SARS-CoV-2 neutralizing activity of mAbs isolated before the surge of Omicron subvariants revealed that mutations in Omicron subvariants such as BA.1, BA.1.1, BA.2, BA.2.12.1, and BA.4/5 contributed to evasion from antibody neutralization (26). Omicron variants, such as BQ.1.1 and XBB, harbored an even greater number of mutations that account for immune evasion (27). We thus tested whether O5C2 broadly recognizes S variants.

**Figure 2:**
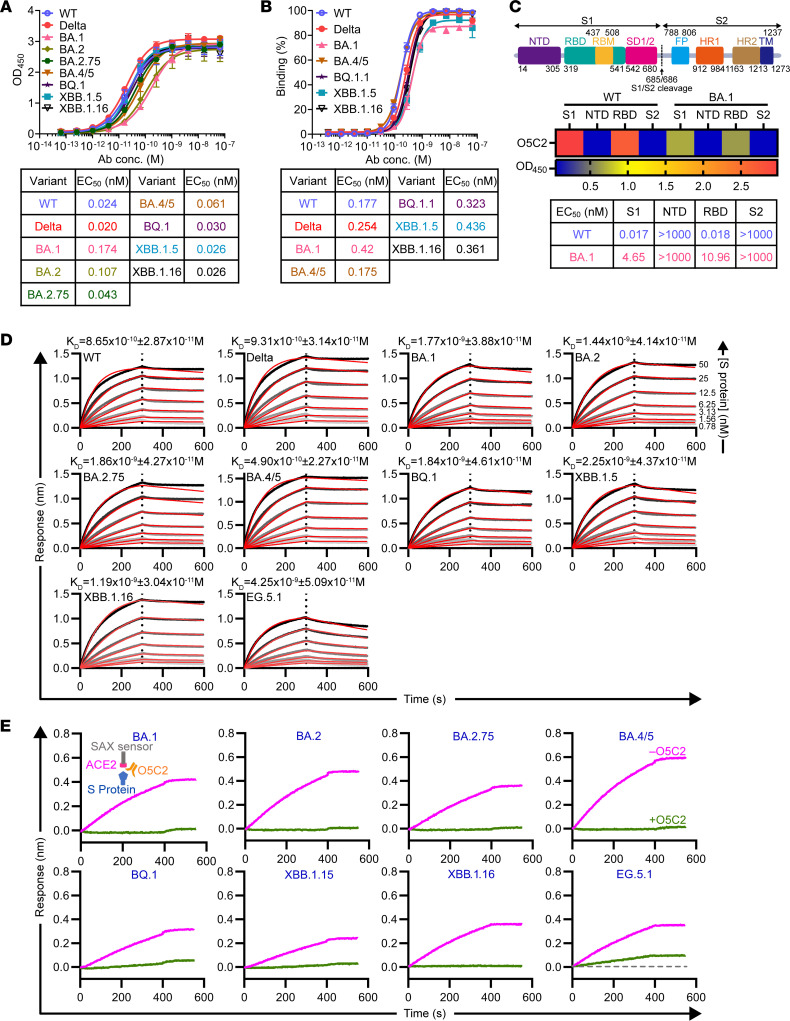
O5C2 mAb broadly recognizes the RBD of S proteins of a range of SARS-CoV-2 strains from WT to Omicron variants and blocks S protein binding to hACE2. (**A**) ELISA showing O5C2 binding to the S ectodomain of the WT strain, and Delta and Omicron variants (top). The EC_50_ (nM) values of O5C2 against various S proteins are indicated (bottom). (**B**) FACS showing O5C2 binding to S-293T cells expressing the S protein of the WT strain and Delta and Omicron variants (top). The EC_50_ (nM) values of O5C2 against various S proteins are indicated (bottom). (**C**) Linear diagram of the sequence/structural domains of WT S protein (top). Heatmap of the OD values from ELISA showing the binding of O5C2 to S1 and the RBD of WT and BA.1 S proteins (middle). The calculated EC_50_ is indicated (bottom). (**D**) BLI (Octet) showing the binding kinetics of O5C2 to the S ectodomain of the WT, Delta and Omicron variants. The *K_D_* values are indicated. (**E**) BLI sensorgrams (SAX sensor) of immobilized ACE2 binding to S proteins from the indicated Omicron variants (magenta) and S proteins preincubated with O5C2 (green). Results in **A** and **C** are the representative results of 3 replicates from 2 independent experiments. Results in **B** are 3 replicates from 1 experiment. Results in **D** are the representative results from 1 experiment. Data in **A** and **B** are presented as the mean ± SEM. The EC_50_ values in **A**–**C** were calculated by fitting a curve using a 4-parameter dose-response nonlinear regression analysis. SD1/2, S1 subdomains 1 and 2.

First, the binding of O5C2 to the S proteins of various SARS-CoV-2 variants was validated by ELISA (Figure 2A) and FACS analysis of various S-293T cells (Supplemental Figure 4 and Figure 2B). Our results verified that O5C2 had broad-spectrum activity, being able to recognize S (sub)variants, including WT, Delta, BA.1, BA.2, BA.2.75, BA.4/5, BQ.1, BQ.1.1, XBB.1.5, and XBB.1.16, at EC_50_ values below nanomolar levels. However, O5C2 mAb did not recognize the S proteins of SARS-CoV-1, Middle East respiratory syndrome coronavirus, and common human coronaviruses (HCoVs), including HCoV-HKU1, HCoV-NL63, HCoV-OC43, and HCoV-229E, as shown by ELISA (Supplemental Figure 5). Furthermore, ELISA showed that O5C2 targeted the RBD of the WT and BA.1 S proteins (Figure 2C). The broad binding activity of O5C2 to S protein was further validated by biolayer interferometry (BLI, Octet) analysis (Figure 2D), which demonstrated that O5C2 bound to a panel of S protein variants, including WT, Delta, BA.1, BA.2, BA.2.75, BA.4/5, BQ.1, XBB.1.5, XBB.1.16, and EG.5.1, with *K_D_* at the nanomolar concentration level.

On the basis of the above finding that O5C2 targets S-RBD, we next investigated whether O5C2 prevents the interaction between hACE2 and S protein. To assess the potential broad-spectrum neutralizing activity of O5C2 against different Omicron sublineages, we used BLI to carry out a competitive binding assay of O5C2 to the S proteins of several Omicron variants, including BA.1, BA.2, BA.2.75, BA.4/5, BQ.1, XBB.1.5, XBB.1.16, and EG.5.1. A GFP-fused ACE2 was first biotinylated and immobilized onto a streptavidin BLI biosensor (SAX). The S protein variants were individually incubated with O5C2 prior to the BLI binding assay. If O5C2 formed a stable complex with the S protein variants in competition with ACE2, the BLI assay would show no kinetic response. Indeed, while the control groups of free S protein variants exhibited the anticipated kinetic traces indicative of ACE2 binding, preincubation with O5C2 completely abolished ACE2 binding for nearly all S protein variants. One exception was EG.5.1, which showed a partial competitive effect. These results suggest broad-spectrum competition for ACE2 binding, regardless of sequence variants within the RBD of the S protein (Figure 2E). Taken together, O5C2 possesses broad-spectrum binding to the RBD of S protein of SARS-CoV-2 variants, including the WT and Omicron variants, which occludes S protein binding to hACE2.

### Structural basis of BQ.1 S protein binding to receptor ACE2 and O5C2.

**Figure 3:**
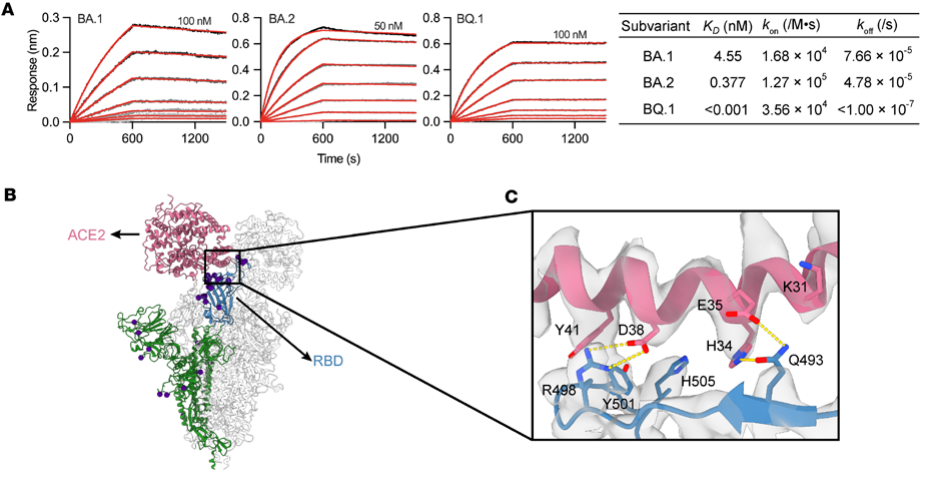
Binding kinetics and structure of trimeric BQ.1 S proteins in complex with hACE2. (**A**) BLI sensorgrams of immobilized ACE2-Fc binding to S protein. BLI sensorgrams of Omicron S protein binding to ACE2-Fc protein, which was immobilized on the sensor tip. The highest concentrations of S protein used in independent BLI binding assays are labeled on the first line with the 2-fold serial dilutions. The kinetic parameters derived from global fitting of the sensorgrams, including the on-rate (*k*_on_), off-rate (*k*_off_), and *K_D_*, are shown below, with the fitted data shown by red lines that are overlaid with the experimental curves. (**B**) Ribbon representation of the BQ.1 S–ACE2 complex, displaying as a trimeric BQ.1 S protein and 2 hACE2 receptors. For clarity, only 1 S protein and 1 ACE2 are colored. ACE2, RBD, and the remaining portion of BQ.1 are shaded in pale violet, steel blue, and forest green, respectively. The mutant residues in BQ.1 are depicted as indigo spheres, representing their Cα atoms. (**C**) Zoomed-in view highlighting the critical interface residues in the RBD of BQ.1 interacting with the α1 helix of ACE2. The electron density map of the BQ.1–ACE2 complex is shown and colored in gray. The yellow dashed lines indicate salt bridge or hydrogen bond interactions.

To further dissect the mechanisms accounting for the disruption of S protein binding to ACE2 by O5C2 (Figure 2E), we next examined the structural basis of the interaction between Omicron S protein and ACE2, as well as that of O5C2 binding to Omicron S protein. In late 2022, the BQ.1 subvariant of Omicron emerged as a predominant strain. We investigated its increased infectivity through comparison of its ACE2 binding affinity to those of the earliest Omicron subvariant (BA.1) and its parental strain (BA.2) using BLI (Figure 3A). The ectodomain of ACE2 was fused to human Fc to form a dimer. Fc-fused ACE2 was immobilized onto the anti-human Fc capture biosensor, resulting in the outward arrangement of ACE2 to minimize steric hindrance resulting from the immobilization procedure. The BQ.1 S protein exhibited the fastest *k*_on_ (3.56 × 10^4^/M•s) and excessively slow *k*_off_ (<1.00 × 10^–7^/s), resulting in an estimated *K_D_* of less than 1 pM.

To investigate the structural basis of the enhanced ACE2 binding by the S protein of BQ.1, we determined the cryo-EM structure of the S protein of BQ.1 in complex with ACE2 (Figure 3B and Supplemental Table 2). Compared with BA.1 and BA.2, BQ.1 carries an R493Q reversion, forming a bipartite hydrogen bonding network with H34 and E35 of ACE2 (Figure 3C and Supplemental Figure 6A). Unlike Q493 in BQ.1, R493 in BA.1 and BA.2 can form only a single hydrogen bond with E35 of ACE2 (Supplemental Figure 6A). Even when compared with the original parental strain, which also features a glutamine residue at position 493 but forms a hydrogen bond only with E35 of ACE2, the bipartite interaction manner of Q493 in BQ.1 indeed contributes to the increased affinity for ACE2. Furthermore, the interaction between R498 in BQ.1 and D38 of ACE2 involves a bidentate salt bridge, which was opposite from the monodentate mode observed in BA.1 and BA.2. This difference may also strengthen the interactions between BQ.1 and ACE2. In addition, the N501Y mutation is implicated in T-shaped π–π stacking with Y41 of ACE2 (Figure 3C). Several residues in the ACE2 contact region underwent substitutions to positively charged amino acids (N440K, T478K, Q498R, and Y505H), which are commonly observed among Omicron subvariants (Supplemental Figure 6B and Supplemental Table 3). Specific mutations (D405N and E484A) also enhance ACE2 interactions by reducing repulsive charges, and mutations K417N and S477N contribute to maintaining a suitable surface potential for ACE2 interactions. These modifications collectively augment ACE2 binding by increasing the positive electrostatic surface potential around the RBM, thereby strengthening its affinity toward the negatively charged RBD binding site of ACE2. Overall, our complex structure provides a rationale for the augmented affinity of BQ.1 for ACE2.

**Figure 4:**
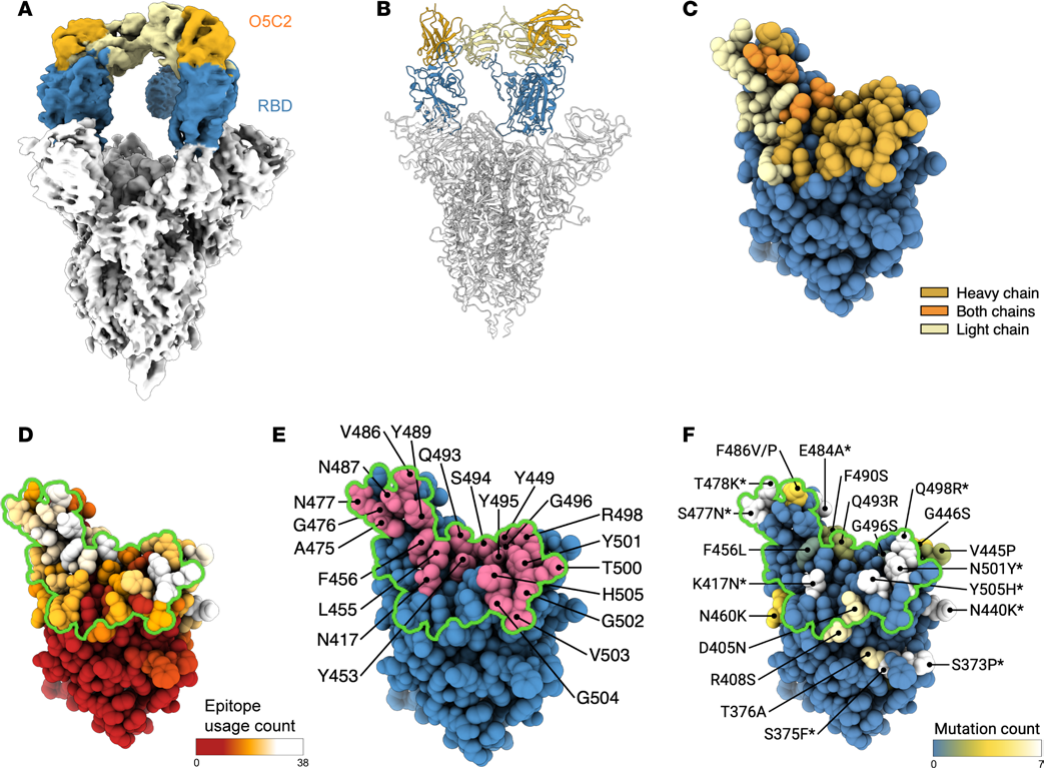
Structural mapping of BQ.1-RBD of the ACE2 and O5C2 binding interface. (**A**) Cryo-EM map and view of the structural ribbon (**B**) of BQ.1 S protein complexed with O5C2. The RBD of BQ.1 S protein is colored blue, and the Fab of antibody O5C2 is colored gold (heavy chain) and yellow (light chain). (**C**) Structural mapping of the binding interface defined as atoms within BQ.1-RBD that are within 5 Å of O5C2: residues of BQ.1-RBD located at the binding interface interacted with O5C2 heavy chain (gold), light chain (yellow), and both heavy and light chains (orange). (**D**) Anti–S protein–RBD antibody epitope hot spots on the RBD of Omicron variants. (**E**) Binding interface defined as atoms within BQ.1-RBD that are within 5 Å of ACE2 (magenta). (**F**) The mutation sites in 7 Omicron variants (BA.1, BA.2, BA.2.75, BA.4/5, BQ.1, XBB.1.5, and EG.5.1). The conserved mutated residues are labeled with asterisks. The O5C2 binding epitope region derived from **C** is colored in green.

We next used the information above regarding the critical residues of ACE2 required to interact with BQ.1 S protein, to determine whether the structural epitope of O5C2 maps to the critical interface between BQ.1 S protein and ACE2. To do this, we performed cryo-EM structure analysis of O5C2 in complex with the S protein of BQ.1 (Supplemental Table 2). The resulting cryo-EM map revealed the asymmetric binding mode of O5C2 to the 3 RBDs of the S protein (Figure 4, A and B). One of the 3 RBDs exhibited a well-defined cryo-EM density corresponding to the Fab of O5C2; another RBD exhibited slightly less resolved cryo-EM density; and the final RBD showed limited and fragmented cryo-EM density that precluded further model building of the Fab (Supplemental Video 1). We focus-refined the cryo-EM map of the RBD in complex with the Fab of O5C2, enabling subsequent model building of the Fab. Compared with the RBM, the structural epitope of O5C2 on the RBD of BQ.1 (Figure 4C) covered an area of 1,019 Å^2^, which was much larger than the RBM of BQ.1 (876 Å^2^). Importantly, the ACE2 binding interface was completely covered within the O5C2 epitope (Figure 4E). O5C2 corresponded to the class I neutralizing antibody against SARS-CoV-2 according to the structure-based classification (28). Furthermore, structural analysis of 84 reported structures of Omicron S protein variants in complex with antibodies showed that the most frequently targeted residues are located within the generic epitope of class I antibodies (Figure 4D and Supplemental Table 4). Indeed, O5C2 targeted the most frequently used structural epitopes of Omicron-specific antibodies, with the exception of N440 and V445 that belong to class 3 antibodies against SARS-CoV-2 (Figure 4F). Although the O5C2 epitope included some variations among the Omicron variants, namely D405N (present only in BA.2 and BQ.1), F486P (present only in BQ.1), and Q493R (present only in BA.1 and BA.2), these mutations are located on the periphery of the epitope and could be tolerated by O5C2 according to the BLI competition assay (Figure 2E).

### O5C2 broadly neutralizes SARS-CoV-2 variants and possesses ADCC.

**Figure 5:**
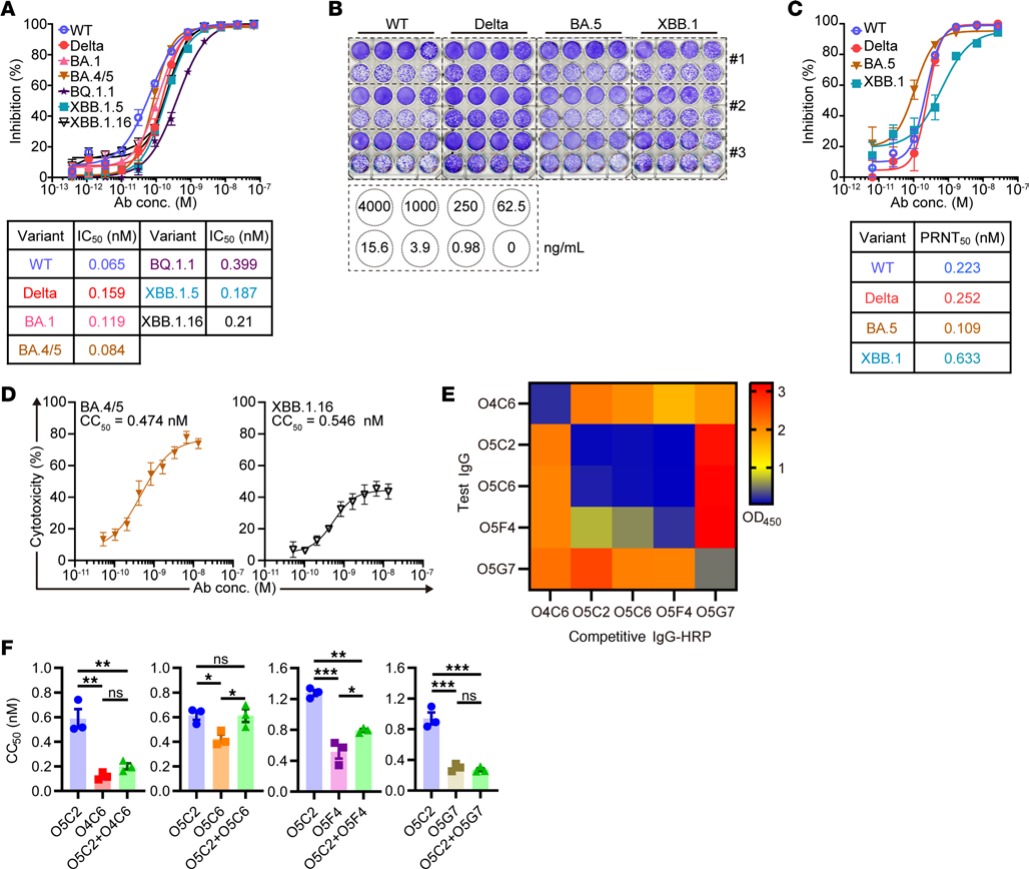
O5C2 possesses pan-neutralizing and broad ADCC activity against SARS-CoV-2 in culture. (**A**) The activity of O5C2 to neutralize the infection of various indicated SARS-CoV-2 pseudotyped viruses. IC_50_ of O5C2 against each variant of SARS-CoV-2 is indicated at the bottom. (**B**) PRNT showing O5C2 effectively neutralizes the infection by the indicated authentic SARS-CoV-2 variants. The concentration of O5C2 used in each well is shown. (**C**) Calculation of the PRNT_50_ of O5C2 against authentic SARS-CoV-2 in **B** is indicated. (**D**) CC_50_ of O5C2 to kill the BA.4/5 (left) and XBB.1.16 (right) S-293T cells by NK-92 MI-FcR cells is shown. (**E**) A heatmap showing the results of competitive ELISA. Color shows the absorbance at 450 nm based on using the indicated HRP-conjugated mAbs (*x* axis) to compete with an unlabeled mAb (10 μg/mL, *y* axis). (**F**) CC_50_ of O5C2 together with another indicated mAb with ADCC activity to kill the BA.4/5 S-293T cells by NK-92 MI-FcR cells. Data in **F** were analyzed by a standard 1-way ANOVA test with Tukey’s multiple comparisons to compare each treatment. Results in **A**, **C**, and **F** are the mean ± SEM, while results in **D** are the mean ± SD (*P < 0.05, **P < 0.01, ***P < 0.001).

On the basis of our structural analysis, which showed that O5C2 occupies several S-RBM residues critical for interacting with ACE2 (Figure 3C and Figure 4E), we next examined whether O5C2 blocks SARS-CoV-2 infection by pseudotyped virus neutralization assays. Notably, O5C2 is able to neutralize a panel of pseudotyped virus–expressing S protein variants, including WT, Delta, BA.1, BA.4/5, BQ.1.1, XBB.1.5, and XBB.1.16, with an IC_50_ below the nanomolar concentration (Figure 5A). Furthermore, O5C2 effectively inhibited the infection of authentic SARS-CoV-2 variants, including WT, Delta, BA.5, and XBB.1, as shown by plaque reduction neutralization tests (PRNTs) (Figure 5B), with the half-maximal concentration of PRNT (PRNT_50_) also below the nanomolar concentration (Figure 5C). These results indicated that O5C2 broadly neutralizes SARS-CoV-2 variants, including various tested Omicron sublineages.

During SARS-CoV-2 infection, the actions of functional antibodies depend not only on blocking viral infection but also on executing their effector function, such as through ADCC via the Fc domain to bind to the FcRs on NK cells (15). Elimination of Fc domain–mediated effector function impaired the protection against SARS-CoV-2 infection mediated by some reported antibodies (29). To evaluate the effect of ADCC triggered by O5C2, the human NK cell line, NK-92 MI, was engineered to stably express CD16 (FcγRIII) (NK-92 MI-FcR) (Supplemental Figure 7A) and then used in an ADCC assay to kill S-293T cells in the presence of mAbs. We found that O5C2 promotes the ADCC of S-293T cells expressing BA.4/5 and XBB.1.16 S protein variants with half-maximal concentration of cellular cytotoxicity (CC_50_) at a lower than nanomolar concentration (Figure 5D). The L234A and L235A (LALA) double mutations at the leucine-leucine residues in the Fc domain caused defective effector functions owing to loss of the interaction between antibody and FcR (30, 31). Indeed, O5C2 harboring the LALA mutation resulted in the loss of ADCC activity against various S-293T cells (Supplemental Figure 7B). In addition to O5C2, 4 other mAbs identified in this study — O4C6, O5C6, O5F4, and O5G7 — possessed ADCC activity toward BA.4/5 S-293T cells (Supplemental Figure 7C).

Previous studies reported that non-neutralizing mAbs interfered with the neutralizing activity of neutralizing mAbs when they targeted the overlapping epitope cluster on SARS-CoV-1 S protein, implying that epitope competition among antibodies may affect their underlying function (32). Therefore, we attempted to test whether other antibodies interfered with the effector function of O5C2. The binding domains of the 15 identified mAbs were examined by ELISA. We found that O5C6, O5F4, and O5G7 are RBD binding mAbs, while O4C6 is an S2 binding mAb (Supplemental Figure 8A). To dissect the epitope recognition of these 5 mAbs with ADCC, we performed competitive ELISA where the tested mAb conjugated with HRP was used to compete with the serially diluted unconjugated mAbs for their binding to BA.4/5 S protein (Supplemental Figure 8B). Our results indicated that O5C2, O5C6, and O5F4 shared some common epitopes (Figure 5E) because O5C6 or O5F4 could completely block the binding of O5C2 to BA.4/5 S. By contrast, O5C2 and O4C6 did not share epitopes, which was consistent with our ELISA result that verified that O4C6 bound to the S2 region. Interestingly, O5G7 appeared to enhance the binding of O5C2 to S protein (Figure 5E). We next analyzed the effect of combining O5C2 with O4C6, O5C6, O5F4, or O5G7 on the ADCC of O5C2 against BA.4/5 S-293T cells (Supplemental Figure 9). We found that in the presence of other mAbs possessing ADCC, regardless of whether they bind to the same epitopes, the ADCC activity of O5C2 was not impeded (Figure 5F). In most cases, the CC_50_ of O5C2 was even markedly reduced (Figure 5F).

Taken together, O5C2 exhibits highly potent neutralization and ADCC against various SARS-CoV-2 variants.

### O5C2 protects against BA.5 infection in K18-hACE2 transgenic mice.

**Figure 6:**
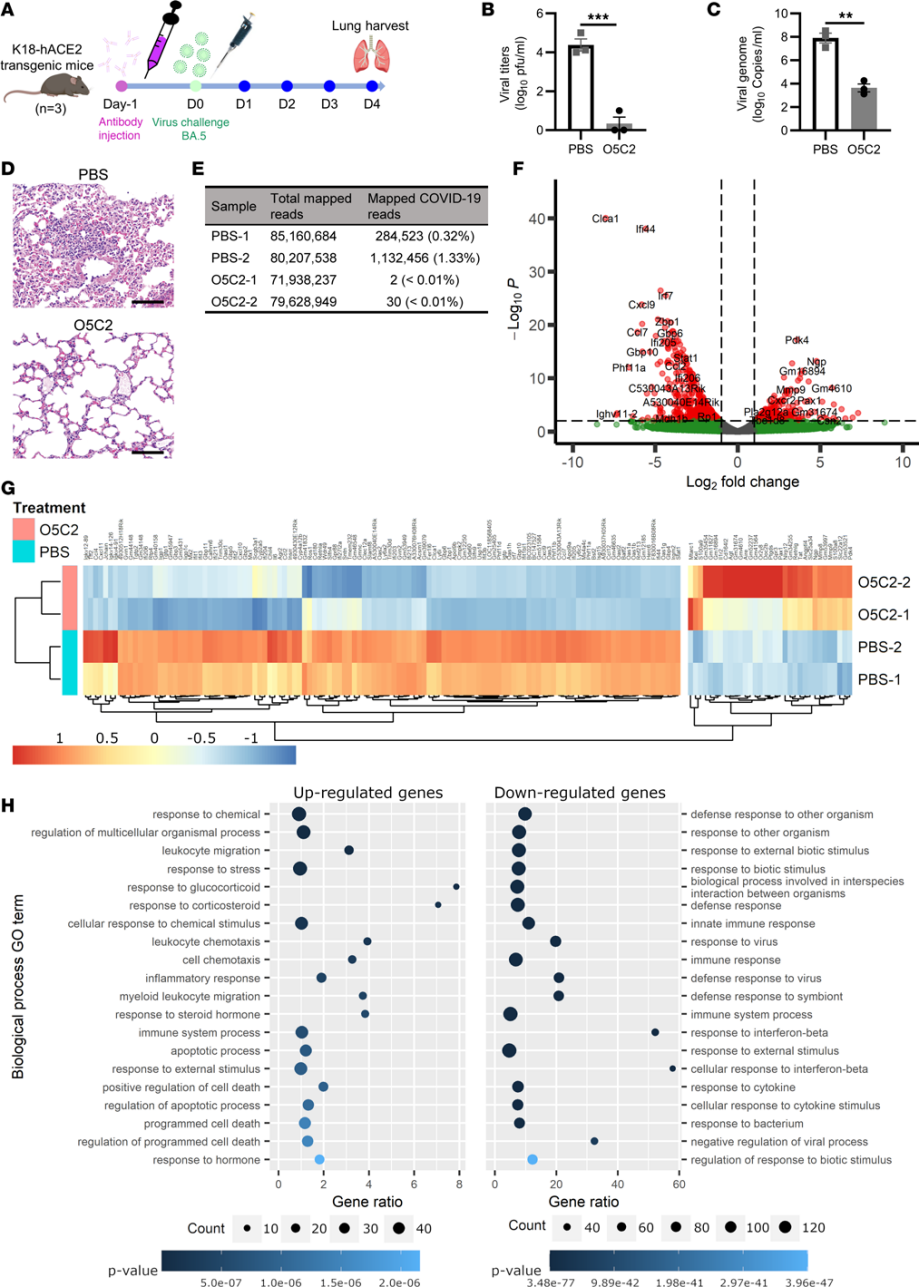
O5C2 protects K18-hACE2 TG mice from BA.5 infection. (**A**) Flowchart of the experimental design of testing the prophylactic activity of O5C2 (15 mg/kg) to protect against challenge with BA.5 SARS-CoV-2 in K18-hACE2 TG mice. (**B** and **C**) Viral levels determined by PRNT (**B**) and real-time quantitative PCR (RT-qPCR) (**C**) in the lungs of infected mice with or without (PBS) pretreatment with O5C2. Data in **B** and **C** were analyzed by the 2-tailed Student’s *t* test to compare the results between treatment with PBS and O5C2 and are presented as the mean ± SEM (**P < 0.01, ***P < 0.001). (**D**) H&E staining showing the histopathology of the lungs in O5C2-pretreated mice on day 4 after BA.5 infection. Scale bar = 100 μm. (**E**–**H**) Transcriptome analysis by RNA-Seq. (**E**) Viral reads identified in the lungs of BA.5-infected mice receiving PBS or O5C2. (**F**) A volcano plot showing the DEGs as red dots, while green and gray dots represent nonsignificant and/or low (log_2_) fold-changes, respectively. The cutoff value for the DEGs was a *P* < 0.05 and log_2_ fold-change ≥ 1. (**G**) A heatmap of significant DEGs. (**H**) Bubble plots showing the top 20 biological process GO terms with the lowest overrepresented *P* value, where the plot on the left is based on upregulated genes, while the right plot is based on downregulated genes. The gene ratio on the *x* axis represents the percentage of genes under a GO term that are differentially expressed. The size of the point indicates the number of DEGs, while the blue color gradient represents the *P* value.

Having demonstrated that O5C2 has broad-spectrum neutralization and ADCC in vitro, we next tested whether O5C2 protects against Omicron infection in vivo. We used K18-hACE2 transgenic (TG) mice as a model of SARS-CoV-2 infection (33). K18-hACE2 mice also permit BA.5 infection but with an attenuated viral load in the lungs, milder pulmonary pathology, and no mortality, compared with those infected with WT SARS-CoV-2 (34). When administered to K18-hACE2 TG mice 1 day before BA.5 intranasal infection (Figure 6A), O5C2 effectively protected K18-hACE2 TG mice from infection (Figure 6, B and C). Low viral titers, determined by PRNT, and reduced RNA levels of BA.5 virus, determined by RT-qPCR, in the lungs at day 4 postinfection were detected in infected mice pretreated with O5C2 (Figure 6, B and C). Lung histology analysis by H&E staining of day 4 postinfected lungs showed that, after infection, PBS-pretreated mice had lung perivascular/interstitial inflammation and endothelialitis (Figure 6D, top), while O5C2-pretreated mice had only limited levels of interstitial inflammation (Figure 6D, bottom).

To understand the underlying mode of protection by O5C2 against BA.5 infection in vivo, we conducted transcriptome analyses of BA.5-infected K18-hACE2 TG mice with O5C2 pretreatment versus PBS pretreatment. We employed bulk RNA-Seq analysis to profile the changes of gene expression in lungs resulting from the interaction among SARS-CoV-2–infected cells and all types of immune cells involved. We took lung paraffin sections from each group of mice to construct concomitant bulk-Seq RNA libraries with rRNA depletion. All 4 libraries yielded an average of 90 million transcript reads, with more than 99% of bases from all reads above the Q30 phred score and only less than 0.01% of reads mapped to rRNA (Supplemental Table 5). In the bulk-Seq transcriptome analyses, the biological replicates all clustered in pairs in the principal component analysis (PCA) plot, and the dot plots showed a high correlation in the PBS-treated group. However, 2 O5C2-pretreated samples showed some distinct features, suggesting a different extent of lung tissue protection by O5C2 in 2 individual mice (Supplemental Figure 10, A and B). The distance matrix (Supplemental Figure 10C) and the heatmap clustering of all expressed genes (Supplemental Figure 10D) also showed a clustering pattern similar to the PCA plot.

We verified that the reads of BA.5 SARS-CoV-2 transcripts were dramatically lower in the O5C2-treated mice compared with the PBS-treated mice after mapping all the transcripts to the BA.5 SARS-CoV-2 reference genome (Figure 6E). Less than 0.01% of the total mapped reads were BA.5 viral reads in the O5C2-treated mice. Next, we conducted differential gene expression analyses comparing BA.5-infected mice with the PBS and O5C2 treatment groups to identify differentially expressed genes (DEGs) associated with the treatment conditions (Supplemental Table 6). Differential gene expression analysis showed that the expression of several genes related to interferon responses, and cytokine and chemokine production, including *Irf7*, *Stat1*, *Ifi44*, *Ifi206*, *Ccl7*, and *Cxcl2*, was reduced in the lungs of infected mice pretreated with O5C2 (Figure 6F). In the cluster heatmap based on differential gene expression, again samples from infected mice treated with PBS and O5C2 formed a distinct clade (Figure 6G). We then conducted Gene Ontology (GO) analysis for GO-enriched categories (Supplemental Table 7). Genes upregulated in the O5C2-pretreated group belonged to GO terms related to programmed cell death and leukocyte migration/chemotaxis (Figure 6H). By contrast, GO terms associated with responses to virus, interferon-β, and cytokines were downregulated in the O5C2-pretreated group (Figure 6H), suggesting that O5C2 ameliorates BA.5-induced immune responses in the lungs. Taken together, our results show the protective effect of O5C2 against BA.5 infection in vivo.

## Discussion

In this study, several mAbs from individuals with breakthrough infections of Omicron subvariants were isolated. One such mAb, O5C2, showing potent binding and neutralizing activities against a broad spectrum of SARS-CoV-2 subvariants at picomolar concentrations, was subjected to further study. O5C2 was found to not only neutralize various SARS-CoV-2 subvariants but also exert effective ADCC. Structural studies revealed that O5C2 can block the ACE2 and S protein interaction by occupying the interface between ACE2 and S protein. RNA-Seq analysis revealed the action of O5C2 in affecting gene expression in the lungs of infected mice. Our findings highlight the potential potency of antibodies to offer broad protection against SARS-CoV-2 infection.

The broadly neutralizing activity of O5C2 can be explained by our structural analysis. We used the S protein of BQ.1 as the model system to examine the molecular basis of its binding to ACE2 and O5C2. Prior to our cryo-EM analyses of the binary complex structures, we used BLI to demonstrate that the S protein of BQ.1 exhibits the highest affinity for ACE2 compared with BA.1 and BA.2, from which BQ.1 is derived. The mutations in the RBD of BQ.1 resulted in an increase in positive charge of the electrostatic surface potential (Supplemental Figure 6B), which is favorable for ACE2 binding. Notably, BQ.1 and BA.2 differ in 5 amino acids within the RBD, specifically, amino acids 444, 452, 460, 486, and 493 (Supplemental Table 3). While T444 is located distant from the RBM, the positively charged R452 of BQ.1 within the RBM positively contributes to ACE2 binding. Q493 of BQ.1 forms bipartite hydrogen bonds with H34 and E35 of ACE2, while R493 of BA.2 and BA.1 forms only 1 hydrogen bond with E35 of ACE2. A recent crystallographic study reported that K460 of BQ.1.1 makes favorable contacts with the N-glycan on N90 of ACE2 (35). Such interactions could not be recapitulated in our cryo-EM structure because of the limited resolution. However, given that BQ.1 and BQ.1.1 only differ in residue 346, which is distant from the RBM, it is likely that the same N-glycan interaction also exists in BQ.1.

The cryo-EM structure of the S protein of BQ.1 in complex with O5C2 revealed a large RBD binding interface that was approximately 17% larger than that of the ACE2 binding interface (Figure 4E). This exceptionally large epitope encompasses 35 residues, whereas the average epitope size of the 19 class I antibodies included in the structural comparison was 21.5 ± 6.5 residues (Supplemental Table 4). Structural mapping of the epitope of O5C2 indicated that the sequence variations in the Omicron variants were located around the periphery of the epitope, and thus the mutations had negligible effects on RBD binding by O5C2. The exceptionally large RBD binding interface that minimizes the negative impacts of sequence variations among the Omicron variants may account for the broad-spectrum neutralizing activity of O5C2 against all Omicron variants tested in this study, including the most recent EG.5 variant.

Neutralizing mAbs against SARS-CoV-2 generally do not require Fc-mediated effector function to provide a prophylactic effect, but the Fc-dependent activity is important for the therapeutic function of mAbs (36, 37). O5C2 possesses potent efficacy against a panel of SARS-CoV-2 subvariants. We postulated that its activity in vivo may depend not only on its neutralizing activity but also on its effector function. It has been reported that the ADCC activity of a mAb does not always correlate with its neutralizing activity and that cross-reactive ADCC effector functions alone may not be sufficient for conferring full protection against SARS-CoV-2 (38). We therefore inferred that the effective ADCC activity of O5C2 can be largely attributed to the high affinity to, and cross-recognition of, S protein, which result from the structural overlapping of the O5C2 binding and ACE2 binding regions against S protein. We also found that another mAb isolated in this study, O5G7, had stronger ADCC activity (Supplemental Figure 9), but weaker neutralizing activity (Figure 1F) against BA.4/5, than O5C2. These results showed that the ADCC and neutralizing activities of a given mAb do not always correlate. It is interesting that when administered with O5C2 and O5G7, the ADCC activity against BA.4/5 is improved compared with O5C2 treatment alone, suggesting that O5C2 and O5G7 may recognize different epitopes on the RBD region (Figure 5E). Our competitive ELISA results also supported this assumption that in the presence of O5G7, O5C2 binds more efficiently to S protein. Therefore, we suspect that the binding of O5G7 to the RBD may further expose the RBD epitope for O5C2, though this requires further structural studies to confirm. Additionally, another RBD-recognizing mAb identified in this study, O5C6, could completely block the binding of O5C2 with S protein, as shown by competitive ELISA (Figure 5F), suggesting that these 2 mAbs shared a similar epitope on S protein. However, unlike O5C2, which can neutralize both BA.4/5 and XBB.1.5, O5C6 was not able to neutralize XBB.1.5 (Figure 1F). Further structural analysis of the O5C6 and S protein complex may reveal the critical mutations on S protein that confer antibody immune evasion from XBB.1.5 infection. Furthermore, the Fc-dependent protective effect of the mAb can be mediated through ADCC, ADCP, and ADCD. ADCP is mostly mediated through FcγRI or FcγRIIA on macrophages or neutrophils (39, 40). The contribution of ADCP and ADCD to the protection by O5C2 remains to be investigated.

The mode of action of protective antibodies in vivo during SARS-CoV-2 infection, particularly at the molecular level, is largely unknown. Using bulk RNA-Seq analysis, we not only validated the dramatic drop in viral RNA reads in O5C2-treated mice but also revealed the pathways induced by O5C2 in vivo. We found that upon BA.5 infection, in the presence of O5C2, genes related to the responses to leukocyte chemotaxis and cell death were upregulated, compared with the PBS-treated group. During SARS-CoV-2 infection, multiple cell death pathways, including apoptosis, necroptosis, ferroptosis, and pyroptosis, were induced in infected cells (41, 42). Because apoptotic cell death is a less deleterious method of eliminating damaged cells, often devoid of inflammation, compared with other types of cell death (43), we assume that the elevated expression of these genes enriched in the programmed cell death category may be relevant to the effector function of the mAb that induced apoptotic cell death of the infected cells (44). Specifically, several genes regulating programmed cell death, or apoptotic processes, including *Cdkn1a*, *Adora1*, and *Agt1*, were upregulated in O5C2-treated mice (Supplemental Table 7). The role of *Adora1* and *Agt1* in SARS-CoV-2 infection and ADCC has not been demonstrated. Previous studies showed that several cell cycle–related genes were dysregulated in coronavirus infection (45, 46). In particular, a study showing COVID-19–specific transcriptomic signatures included *Cdkn1a* (47). Combined with our results indicating the protective effect of O5C2, we hypothesized that elevated expression of *Cdkn1a* (p21), the cell cycle inhibitor (48), may be one of the host defense mechanisms to eliminate SARS-CoV-2. Additionally, several genes, such as *Cxcr1*, *Cxcr2*, and *Il1r2* (Supplemental Table 7), in the regulation of leukocyte chemotaxis and migration, were upregulated in the lungs of O5C2-treated mice after BA.5 infection. Consistent with our histopathology results, O5C2-treated mice bear less lung inflammation as the levels of *Il1r2*, encoding the anti–IL-1–mediated inflammation molecule (49), increased. Cxcr1 and Cxcr2 and their ligands, CXCL1/CXCL2, are involved in recruiting neutrophils and macrophages (50, 51). Because neutrophils and macrophages account for the effector functions of antibodies, upregulation of Cxcr1 and Cxcr2 can also explain the enhanced effector functions in O5C2-treated mice.

By contrast, our RNA-Seq analysis showed that several genes involved in the responses to virus and interferon-β were downregulated in the lungs of O5C2-treated mice after BA.5 infection. These genes included *Irf7*, *Stat1*, *Il6*, *Il33*, and other interferon-responsive genes. Imbalanced interferon responses have been shown to contribute to the pathology of COVID-19 (52). IRF7 transactivates type I interferon genes (53). During viral infections in the respiratory tract by influenza A virus, the attenuated activity of IRF7 can ameliorate acute lung injury (54). In response to interferon signals, STAT1 is activated and regulates many genes involved in antiviral responses. Increased STAT1 expression and activation were found in severe COVID-19 (55). Furthermore, pro-inflammatory cytokine levels of IL-6 and IL-33 are associated with acute SARS-CoV-2 infection and severe COVID-19 (56, 57). Taken together, the downregulation of genes in response to the virus and interferon-β explains the effect of O5C2 in vivo, which is not only to neutralize BA.5 viral loads, resulting in a reduced response to the virus, but also to alleviate inflammatory responses resulting from viral infection.

In summary, using VLPs and single–B cell screening, we isolated many mAbs from donors with breakthrough infection. These mAbs showed cross-recognition of a broad spectrum of SARS-CoV-2 strains, particularly the Omicron subvariants. In particular, mAb O5C2 offered broad protection against SARS-CoV-2 infection because it bound to the virus with high affinity, sterically hindering the binding of ACE2 to S protein. O5C2 also affects the expression of genes important for programmed cell death, leukocyte chemotaxis, antiviral activity, and interferon responses in lung tissues after Omicron BA.5 infection. Our findings not only provide important insights into how broadly protective mAbs function in vivo but also highlight the potential for designing new pan-protective mAbs against SARS-CoV-2 through antibody engineering.

## Methods

### Sex as biological variable.

Sex was not considered as a biological variable.

### Cell lines and culture medium.

Vero-E6 cells (American Type Culture Collection [ATCC], CRL-1586), HEK293T cells (ATCC, CRL-3216), HEK293T cells expressing human ACE2 (hACE2-293T), and HEK293T cells expressing S proteins on the surface (S-293T) were cultured in DMEM (Gibco, 11965-092) containing 10% (v/v) FBS (Gibco, 10437-028), 0.055 mM 2-ME (Gibco, 21985-023), and 1% penicillin and streptomycin (P/S) (Gibco, 15140122) in an incubator at 5% CO_2_ at 37°C. NK-92 MI-FcR cell line (Bioresource Collection and Research Center) was maintained in the α-MEM without nucleoside complete medium (Gibco, 12561-049) containing 12.5% FBS, 12.5% horse serum (Gibco, 26050-088), 0.1 mM 2-ME, 0.2 mM inositol (MilliporeSigma, i7508), 0.02 mM folic acid (MilliporeSigma, F8758), and 1% P/S in an incubator at 5% CO_2_ at 37°C. Expi293F cells (Thermo Fisher Scientific) were cultured in the Expi293 Expression Medium (Gibco, A1435102), or on an orbital shaker (125 rpm), at 37°C in 8% CO_2_. Mycoplasma testing was performed by using the EZ-PCR Mycoplasma Detection Kit (Sartorius, 20-700-20).

### Cell sorting of S protein and SARS-CoV-2 VLP-specific human B cells.

The Research Ethics Committee of Academia Sinica approved the research project to obtain peripheral blood of donors who had recovered from COVID-19. PBMCs were isolated from recovered donors from June 2022 to July 2023, using Ficoll and centrifugation at 700*g* for 30 minutes at room temperature. Human B cells were then purified using CD19 MicroBeads (Miltenyi Biotec, 130-050-301). The isolated cells were costained with BA.1 S protein at 20 μg/mL and EGFP-N-BA.4/5-S-VLP in PBS by rotating at 4°C for 30 minutes. The cells were then washed with PBS twice, followed by staining with diluted APC anti-His Tag antibody (BioLegend, 362605; 1:200). After 1 PBS wash, the cells were resuspended in cold PBS containing diluted propidium iodide (BioLegend, 421301; 1:400). Live, single S protein^+^/VLP^+^ B cells were sorted into a 96-well PCR plate (Applied Biosystems, 4346907) with 10 μL of catch buffer (10 mM Tris-HCl, pH 8, and 5 U/μL RNasin from Promega, N2518) at 1 cell/well by BD FACSAria II. After sorting, the plates were stored at −80°C.

### Cloning of human mAb.

Ig genes from a single B cell were isolated following previously described methods (58, 59). Briefly, the sorted B cells were used for subsequent RNA reverse transcription (Applied Biosystems, 4368813). After sequencing, the sequences of the nested PCR products were analyzed using IMGT/V-Quest (http://www.imgt.org) to determine the highest homology gene loci of the germline V, D, and J genes. The identified candidate IgH and IgL cDNA segments were then subcloned into a chimeric Ig expression vector. This vector was a modification of the tandem chimeric antibody expression vector and the pIgG1(κ) vector (provided by T.W. Chang, Academia Sinica, Taipei, Taiwan). LALA mutations at the Fc region of the mAb were generated by site-directed mutagenesis of cDNA, substituting 2 leucine residues with 2 alanine residues at amino acid positions 234 and 235.

### Pseudotyped SARS-CoV-2 neutralization assay.

All the experiments were performed at the National RNAi Core Facility (Academia Sinica, Taipei, Taiwan). The hACE2-293T cells, at a density of 1 × 10^4^/well, were seeded into 96-well, white plates (Corning Costar) and cultured at 37°C for 24 hours. The next day, serially diluted mAbs or plasma were incubated with 1,000 transducing units/well of various pseudotyped SARS-CoV-2 variants expressing luciferase in a 96-well plate at 37°C for 1 hour, as described previously (60). The seeded hACE2-293T cells were then treated with the mixtures of mAbs and pseudotyped virus at 37°C for 24 hours. The supernatants were then replaced with new medium, and the cells were further cultured for 48 hours. Each well was then incubated for 3 minutes with ONE-Glo luciferase reagent (Promega) for cell lysis and luminescence analysis. The relative light units (RLUs) were quantified using a microplate spectrophotometer (Molecular Devices). The percentage inhibition of the mAb is defined as the percentage decrease in RLUs compared with the RLUs in wells without mAb treatment after subtraction of the value for the background wells containing cells only (no virus). The IC_50_ was calculated by nonlinear regression of the percentage inhibition of the serially diluted mAb using GraphPad Prism software (version 10.0.3).

### Cryo-EM sample preparation and data collection.

Three microliters of BQ.1 S protein were mixed with 1.2 equivalent of sfGFP-ACE2 or mAb (O5C2) (BQ.1-S protein:sfGFP-ACE2 or BQ.1-S protein:O5C2), and the mixture was incubated at room temperature for 1 hour and concentrated to 1.5 mg/mL for cryo-EM grid preparation. The complexed protein samples were applied onto a 300-mesh Quantifoil R1.2/1.3 holey carbon grid. The grids were glow-charged at 20 mA for 20 seconds. After 30 seconds of incubation, the grids were blotted for 2.5 seconds at 4°C with 100% humidity and vitrified using a Vitrobot Mark IV (Thermo Fisher Scientific). Data acquisition was performed on a 300 keV Titan Krios microscope equipped with a Gatan K3 direct detector in the super-resolution mode using EPU 2.10 software (Thermo Fisher Scientific). Videos were collected at a defocus range of −0.8 to −1.8 μm at an original magnification of 81,000×, resulting in a pixel size of 0.55 Å. A total dose of 50 e/Å^2^ was distributed over 50 frames with an exposure time of 1.8 s. The data set was collected with an energy filter (slit width 15–20 eV), and the dose rate was adjusted to 8 e/pixel/s.

### Model building and refinement.

An initial model of BQ.1-S protein:sfGFP-ACE2 was generated based on Protein Data Bank (PDB) entry 8DM5 (61) using Swiss-Model (62). The coordinate was divided into individual domains and manually fitted into the cryo-EM map using UCSF ChimeraX v.1.5 (63). A similar approach was used for BQ.1-S protein:O5C2, for which the antibody template structure (PDB: 7LKA) (64) was generated using Swiss-Model as the initial model. After iterative refinements, the coordinate was further processed by real-space refinement in Phenix v.1.20.1 (65). The final models were assessed by MolProbity (66). Structural visualization and rendering of structural representations were achieved using ChimeraX and Pymol v.2.4.1 (Schrodinger Inc.).

### Bioinformatics analysis of epitope usage of anti-Omicron antibodies.

Statistics of residue-specific epitope usage of anti-Omicron antibodies was carried out by using 84 protein structures of antibody-bound S proteins of Omicron variants deposited in the PDB as of July 31, 2023 (Supplemental Table 4). Structural epitopes were defined as the residues within the RBD that are within 5 Å of any atom of the antibodies calculated by ChimeraX v.1.5. The list of epitope residues was compiled and used to generate a hot spot map to illustrate the epitope usage frequency by ChimeraX.

### ADCC assay.

S-293T cells were washed twice with PBS. Cells were resuspended in 5 μM Calcein-AM (Invitrogen, C3099) solution (in PBS) and incubated at 37°C for 20 minutes. Cells were then washed 3 times with PBSF (1× PBS, pH 7.4, containing 5% FBS, 1% P/S), adjusted to 1 × 10^5^ cells/mL, and seeded into 96-well, U-bottom plates at 1 × 10^4^ cells/well (100 μL/well). Anti–SARS-CoV-2 mAbs (10 μL/well) and InVivoMAb human IgG1 isotype control (BioXCell, BE0297) at various diluted concentrations were added to the wells. Subsequently, NK-92 MI-FcR cells (1.12 × 10^6^ cells/mL in PBSF) were added to each well (90 μL/well at the effector/target ratio = 10:1) and incubated for 4 hours. The “maximum-release” well received 100 μL of lysis buffer (2% Triton X-100 in PBSF) in place of the antibody and NK-92 MI-FcR cells. The plates were centrifuged at 600*g* for 3 minutes at room temperature, and 150 μL of the supernatant was transferred to a black, 96-well, F-bottom plate. ADCC was assessed according to the release of calcein fluorescence. The fluorescence (excitation: 485 nm and emission: 535 nm) was measured using the SpectraMax iD5 (Molecular Devices). The cytotoxicity percentage was determined using the following formula: % specific lysis = ([F^ET^ – F^Spontaneous^ ^release^]/[F^Max^ ^release^ – F^Spontaneous^ ^release^]) × 100%.

F^Max^ ^release^ is fluorescence intensity with lysed target cells. F^Spontaneous^ ^release^ is fluorescence intensity with target cells only. F^ET^ is fluorescence intensity with effector cells, target cells, and anti–SARS-CoV-2 mAbs.

### Challenge of K18-hACE2 mice with SARS‑CoV‑2 BA.5.

To evaluate the therapeutic potency of mAbs, K18-hACE2 mice (purchased from The Jackson Laboratory) were subjected to SARS-CoV-2 BA.5 infection (67). Each mouse was intraperitoneally injected with mAb O5C2 (15 mg/kg) or PBS, then, 24 hours later, was intranasally inoculated with 10^4^ PFU SARS-CoV-2 BA.5 (hCoV-19/Taiwan/TSGH-8189/2022, provided by the Taiwan Centers for Disease Control and Prevention). To determine the lung viral titers of SARS-CoV-2 BA.5–infected mice, the mice were euthanized for the collection of lung tissues at day 4 postinoculation. One half of the lung tissue (right lobe) was homogenized, and the supernatant was used for a plaque assay and viral quantitative PCR, according to a previously published protocol (68). The left lobe of the lung tissue was harvested for histopathological H&E staining, as previously described (69). All work with infectious SARS-CoV-2 was performed in Institutional Biosafety Committee–approved BSL3 and A-BSL3 facilities at the National Defense Medical Center. Animal studies were carried out in accordance with the recommendations in the Animal Research: Reporting of In Vivo Experiments guidelines.

### Statistics.

All results in this study are presented as the mean ± SEM or SD as indicated. The data in Figure 5F were analyzed by a standard 1-way ANOVA test with Tukey’s multiple comparisons to compare each treatment. Data in Supplemental Figure 7B were analyzed by a 2-tailed Student’s *t* test to compare the results between treatment with O5C2 and O5C2-LALA. In Figure 6, B and C, data were analyzed by a 2-tailed Student’s *t* test to compare the results between treatment with PBS and O5C2. Curves were fit in nonlinear regression using GraphPad Prism software (version 10.0.3). *P* < 0.05 was considered to indicate statistical significance.

All other methods are described in the Supplemental Methods.

### Study approval.

The IRB of Academia Sinica approved the research project to obtain peripheral blood of donors who had recovered from COVID-19. The written informed consent was obtained from each donor before sample collections. The protocols of the animal study were approved by the IACUC of the National Defense Medical Center and Academia Sinica.

### Data availability.

The atomic coordinates of ACE2- and O5C2-bound S-BQ1 generated in this study have been deposited in the PDB under the accession codes 8XAL and 8XBF, respectively. The cryo-EM map of ACE2- and O5C2-bound S-BQ1 have been deposited in the Electron Microscopy Data Bank under the accession codes EMD-38201 and EMD-38216, respectively. The bulk RNA-Seq data set of control and O5C2-treated *M*. *musculus* lung tissue samples are deposited in Sequence Read Archive section of NCBI (BioProject number: PRJNA1049508; accession numbers: SRR27118421–SRR27118424). Values for all data points in graphs are included the Supporting Data Values file.

The code used for differential gene expression analysis related to bulk RNA-Seq data can be accessed through GitHub (https://github.com/danielylup/O5C2_CovidMouseLung; commit ID 2148c74).

## Author contributions

KIL conceived the research. MYJL, STDH, and KIL supervised the research. YH Chang, WNC, FJC, HYT, CPT, JHY, and YK isolated B cell clones and characterized mAbs. MFH, MHW, and YC Chang performed cryo-EM structure determination. CHH, LYB, AYC, and CCC bred the animals and performed virus challenge animal studies in an A-BSL3 facility. YC Chou, CCL, CSC, JJL, and YLL conducted pseudotyped virus experiments or authentic virus neutralization in a BSL3 facility. LYC and TA generated engineered NK-92 MI-FcR cells. WLK and YH Chen performed RNA-Seq and data analysis. YH Chang, MFH, STDH, and KIL wrote the paper.

## Supplementary Material

Supplemental data

Supplemental table 1

Supplemental table 4

Supplemental table 6

Supplemental table 7

Supplemental video 1

Supporting data values

## Version 1 05/22/2024

Electronic publication
