# Supplementary material for: Functional and structural investigation of a broadly neutralizing SARS-CoV-2 antibody
Source: JCI Insight. 2024 May 22;9(10):e179726. doi: 10.1172/jci.insight.179726 (PMC11141937; doi:10.1172/jci.insight.179726)
Supplement: Supplemental data [file jciinsight-9-179726-s259.pdf]

Supplemental information

Functional and structural investigation of a broadly neutralizing SARS-CoV-2 antibody

Yi-Hsuan Chang[1,2,#], Min-Feng Hsu[3,#], Wei-Nan Chen[1,##], Min-Hao Wu[3,##], Wye Lup Daniel Kong[4,##], Mei-Yeh Jade Lu[4], Chih-Heng Huang[5,6,7], Fang-Ju Chang[1], Lan-Yi Chang[3], Ho-Yang Tsai[1,2], Chao-Ping Tung[1], Jou-Hui Yu[1], Yali Kuo[8], Yu-Chi Chou[8], Li-Yang Bai[1], Yuan-Chih Chang[3,9], An-Yu Chen[5], Cheng-Cheung Chen[5,6], Yi-Hua Chen[4], Chun-Che Liao[8], Chih-Shin Chang[8], Jian-Jong Liang[10], Yi-Ling Lin[8,10], Takashi Angata[2,3], Shang-Te Danny Hsu[2,3,11]* and Kuo-I Lin[1,8,*]

1. Genomics Research Center, Academia Sinica, Taipei, Taiwan
2. Institute of Biochemical Sciences, National Taiwan University, Taipei, Taiwan
3. Institute of Biological Chemistry, Academia Sinica, Taipei, Taiwan
4. Biodiversity Research Center, Academia Sinica, Taipei, Taiwan
5. Institute of Preventive Medicine, National Defense Medical Center, Taipei, Taiwan
6. Graduate Institute of Medical Sciences, National Defense Medical Center, Taipei, Taiwan
7. Department of Microbiology and Immunology, National Defense Medical Center, Taipei, Taiwan
8. Biomedical Translation Research Center (BioTReC), Academia Sinica, Taipei, Taiwan
9. Academia Sinica Cryo-EM Center, Academia Sinica, Taipei, Taiwan
10. Institute of Biomedical Sciences, Academia Sinica, Taipei, Taiwan
11. International Institute for Sustainability with Knotted Chiral Meta Matter (WPI-SKCM[2]), Hiroshima University, 1-3-1 Kagamiyama, Higashi-Hiroshima, Hiroshima, Japan

Supplementary Materials

Supplementary References

Supplemental Table
Supplemental Table 1. Binding of 114 cloned mAbs, produced by mini-scale culture, with WT, Delta, BA.1 and BA.4/5 S-293T cells
Supplemental Table 2. Cryo-EM data collection, refinement and validation statistics
Supplemental Table 3. Mutations in the RBD of Omicron S proteins
Supplemental Table 4. Structures of antibody-bound S proteins of Omicron variants deposited in Protein Data Bank.
Supplemental Table 5. The high-throughput sequencing and mapping matrices of the four BA.5 infected mice that were pre-treated with PBS or O5C2.
Supplemental Table 6. List of differentially expressed genes (DEG) between antibody and PBS treatment in infected mouse lung samples.
Supplemental Table 7. List of DEG for each over-represented GO term.

Supplementary Video

Supplementary Video 1. The motion of flexible RBDs of S protein from cryo-EM images.

Supplementary Figure

Supplemental Figure 1. Generation of BA.4/5 S protein expressing VLP

Supplemental Figure 2. A schematic of SARS-CoV-2 Omicron subvariant evolution, indicating the mutations on the RBD

Supplemental Figure 3. Vaccination history of three donors, the neutralizing activity of their plasma against BA.4/5-pseudotyped virus, and the percentage of peripheral blood B cells targeting both BA.1 S protein and eGFP-N-BA.4/5-S-VLP

Supplemental Figure 4. Generation of S-293T cells expressing S proteins from various SARS-CoV-2 variants

Supplemental Figure 5. O5C2 mAb does not recognize S proteins of non-SARS-CoV-2 human coronaviruses

Supplemental Figure 6. ACE2-interacting region in the RBD of S proteins

Supplemental Figure 7. ADCC killing assays reveal that O5C2 has a broad spectrum of ADCC activity and that five mAbs in this study possess ADCC

Supplemental Figure 8. Identification of the domains of S proteins targeted by 15 mAbs by ELISA and competitive ELISA

Supplemental Figure 9. O5C2 works in concert with other mAbs in ADCC

Supplemental Figure 10. Distinct gene expression cluster in infected mice treated with PBS or O5C2

Supplemental Materials

Construction of DNA plasmids expressing full-length S protein or eGFP-tagged N protein

To clone the plasmid enabling simultaneous expression of surface S protein and intracellular fluorescent protein, the DNA cassette, encoding eGFP or mNeptune2.5 tagged with P2A-containing peptide (amino acid sequence is GSGATNFSLLKQAGDVEENPGPAPVAT) at N-terminus, was amplified by PCR and inserted into the multiple cloning site (MCS) of pcDNA6 (Invitrogen, V22120) to generate the pcDNA6-P2A-eGFP or pcDNA6-P2A-mNeptune2.5. The human-codon-optimized DNA fragment of full-length WT S protein (Wuhan/WH01/2019 strain) or S variants with mutations listed below was PCR-amplified from CMV promoter-driven S protein expressing plasmids synthesized from Genomics Inc (Taiwan) or kindly provided by the National RNAi Core Facility (Academia Sinica, Taiwan). Delta S protein includes mutations of T19R, 156-157del, R158G, L452R, T478K, D614G, P681R and D950N as compared to WT S protein. BA.1 S protein includes mutations of A67V, 69-70del, T95I, G142D, 143-145del, 211del, L212I, ins214EPE, G339D, S371L, S373P, S375F, K417N, N440K, G446S, S477N, T478K, E484A, Q493K, G496S, Q498R, N501Y, Y505H, T547K, D614G, H655Y, N679K, P681H, N764K, D796Y, N856K, Q954H, N969K, and L981F as compared to WT S protein. BA.4/5 S protein includes mutations of T19I, L24S, 25-27del, 69-70del, G142D, V213G, G339D, S371F, S373P, S375F, T376A, D405N, R408S, K417N, N440K, L452R, S477N, T478K, E484A, F486V, Q498R, N501Y, Y505H, D614G, H655Y, N679K, P681H, N764K, D796Y, Q954H and N969K as compared to WT S protein. BQ.1.1 S protein includes R346T, K444T and N460K as compared to BA.4/5 S protein. XBB.1.5 S protein includes mutations of T19I, L24S, 25-27del, V83A, 144del, G142D, H146Q, Q183E, V213E, G252V, G339H, R346T, L368I, S371F, S373P, S375F, T376A, D405N, R408S, K417N, N440K, V445P, G446S, N460K, S477N, T478K, E484A, F486P, F490S, Q498R, N501Y, Y505H, D614G, H655Y, N679K, P681H, N764K, D796Y, Q954H, and N969K as compared to WT S protein. XBB.1.16 S protein includes mutations of E180V and K478R compared to XBB.1.5 S protein. EG.5.1 S protein includes mutations of Q52H, F456L and Q613H as compared to XBB.1.5 S protein. The S cDNA (full length) fragment was then cloned into the cloning site preceding P2A-eGFP or -mNeptune2.5 DNA cassette to generate pcDNA6/S-P2A-eGFP or pcDNA6/S-P2A-mNeptune2.5.

To construct the plasmid encoding N-terminal eGFP-tagged N protein, the megaprimer containing the DNA fragment of eGFP conjugated with C-terminal peptide linker with GGS amino acid sequence, and the first 15 nucleotides of N gene was amplified by PCR. This megaprimer along with the primer including the last 18 nucleotides of N gene and stop codon were used to amplify the DNA cassette of N-terminal eGFP-tagged N protein from the plasmid (SARS-CoV-2/Wuhan/7/2020, NCBI reference sequence: NC_045512.2) kindly provided by Dr. An-Suei Yang (Academia Sinica, Taiwan) by PCR. The DNA cassette was then cloned into the multiple cloning sites of pcDNA6.

Generation of VLP expressing eGFP-tagged N and BA.4/5 S protein

Plasmids encoding SARS-CoV-2 E and M protein were purchased from Sino Biological Inc (E: VG40609-UT, M: VG40608-UT). A total of 25 μg of four plasmids expressing full-length BA.4/5-S, E, M or N-terminal eGFP-tagged N protein, respectively, at a molar ratio of 2:3:3:3 (6.25 μg : 6.25 μg : 6.25 μg : 6.25 μg) was mixed and co-transfected into 3×10^6 Expi293F cells in 30 mL of medium with ExpiFectamine™ 293 Transfection Kit (Gibco, A14525). The cells were cultured at 37°C on an orbital shaker (125 rpm) in 30 mL of medium. Cell culture was treated with Enhancer 1 + 2 provided by the transfection kit next day, followed by

additional 2-day culture. The cell culture was then added with 5 mM EDTA to prevent further furin cleavage, followed by centrifugation at 2000 × g for 10 min. The supernatants were collected and filtered with 0.45 μm bottle-top vacuum filter. The filtered supernatants were concentrated 20-fold by 300 kDa MWCO Vivaspin® 20 Centrifugal Concentrator Polyethersulfone (SARTORIUS, VS2051), followed by dialysis against PBS with 1000 kDa MWCO Biotech CE Dialysis Trial Kit (Repligen, 131486T). The eGFP-tagged N and BA.4/5 S protein-expressing SARS-CoV-2 VLPs (eGFP-N-BA.4/5-S-VLP) were snap-frozen in liquid nitrogen and stored at −80°C.

For assessing the binding of eGFP-N-BA.4/5-S-VLP with human ACE2, the hACE2-293T cells were stained with VLP on ice for 30 min, washed with PBS twice and resuspended in PBS. The $eGFP^+$ cells were measured by flow cytometric analysis (BD FACSCanto II), and the results were analyzed by FlowJo (version 10.5.0). To verify the expression of S, E, M and N-terminal eGFP-tagged N proteins by VLP, the VLP was boiled in sample buffer at 95°C for 10 min and run on gradient SDS-PAGE (TOOLS, TFU-GG420), followed by immunoblotting analysis using the following primary antibodies: anti-SARS-CoV-2 spike glycoprotein S2 antibody (Abcam, ab283913), anti-SARS-CoV-2 envelope protein antibody (Cell signaling, 74698), anti-SARS-CoV-2 membrane protein (Cell signaling, 32904) and anti-SARS-CoV Nucleoprotein / NP antibody (Sino Biological, 40143-R001. Horseradish peroxidase (HRP)-conjugated secondary antibodies used here are: light chain specific-peroxidase AffiniPure goat anti-mouse IgG (Jackson ImmunoResearch Inc, 115-035-174) and light chain specific-peroxidase IgG fraction monoclonal mouse anti-rabbit IgG (Jackson ImmunoResearch Inc, 211-032-171).

Fluorescent spectroscopy

Glass slides were cleaned up with 2% Extran® MA 02 (Supelco, 107553), 1N HCl and 70% Ethanol. After drying up, the slides were coated with 1μg/ml poly-L-lysine (Sigma, P6282) in PBS for 30 min at 37°C and washed with PBS for three times. hACE2-293T or HEK293T cells were seeded on slides at 2.5×10^4 cells/cm^2 overnight, followed by wash with cold DMEM to remove unbound cells. The cells were treated with DMEM containing eGFP-N-BA.4/5-S-VLP and incubated on ice for 1hr. The cells were washed with cold PBS for three times and fixed with 4% paraformaldehyde for 1 hr, followed by washed with with PBS and incubated with TrueBlack® Lipofuscin Autofluorescence Quencher for 30 sec (Biotium, 23007). The cells were then washed with PBS and stained with 0.2 μg/ml DAPI (Thermo Scientific, 62248) for 30 min. The PBS-washed cells were mounted with Fluoromount-G® (SouthernBiotech, 0100-01) for confocal imaging. Images were captured by Leica TCS SP8X White Light Laser Confocal Super-resolution Microscope which was equipped with 100 × 1.40 NA oil objective (Leica, 56372). The cells in single field were imaged as Z stack with 10 sections within 0.3 μm and the images of single field were merged by Leica Application Suite X (version 3.5.2.18963).

Human monoclonal antibody production and purification.

For "mini-scale" mAb production, 1.2 μg of IgH expressing and 1.8 μg of IgL expressing plasmids were co-transfected into 3×10^5 Expi293F cells in 3 mL of medium using the transfection kit described above. The next day, cell culture was treated with Enhancer 1+2, with an additional 4-day culture. The supernatants of cell culture were then collected for flow cytometric analysis to screen their binding with S protein. For "midi-scale" mAb production, 12 μg of IgH expressing and 18 μg of IgL expressing plasmids were co-transfected into 3×10^6 Expi293F cells in 30 mL of medium, and Enhancer 1 + 2 was added to the culture next

day. The supernatants from 6-day cultures were harvested and purified by Monofinity A Resin (GenScript, L00433-200). Purified mAb was concentrated and buffer-exchanged into PBS using Vivaspin®6 30 kDa MWCO (cytiva, 28932317). The heat-denatured mAb was subjected to gradient SDS-PAGE (TOOLS, TFU-GG420), followed by Coomassie blue staining using InstantBlue® Coomassie Protein Stain (abcam, ab119211) to confirm the purity.

Establishment of HEK293T cell lines stably expressing S proteins

The pcDNA6/S-P2A-eGFP or -mNeptune2.5 plasmid was transfected into HEK293T cells using Lipofectamine™ 3000 transfection reagent (Invitrogen, L3000001). Transfected cells were treated with blasticidin at 10 μg/mL for selection for 2 to 3 weeks, followed by cell sorting with FACS Aria II to obtain $eGFP^{+}$ or $mNeptune2.5^{+}$ cells. The purified cells were then cultured in DMEM containing 10% FBS and blasticidin (InvivoGen, ant-bl-1) at 10 μg/mL until 90% confluency. The single cells expressing $eGFP^{+}$ or $mNeptune2.5^{+}$ at top 2% levels were then individually sorted into 96-well flat bottom culture plates. After two weeks in culture, the 293T cell line stably expressing S protein was selected from the well that has the most cells elicited fluorescence, as inspected using inverted cell culture fluorescence microscope (Olympus, IX71).

FACS analysis examining the binding of mAbs with surface S protein expressing HEK293T cells

1×10^{5} of S-293T cells were incubated with serially-diluted human mAb, starting from 10 μg /ml, in FACS buffer or supernatants from IgH and IgL plasmids transfected cell culture for 1 hr on ice. The cells were then washed with FACS buffer for three times, followed by staining with 1:200 diluted BV421-mouse anti-human IgG (BD Biosciences, 562581) for 20 min on ice. The cells were then washed with FACS buffer twice. The percentage of cells binding with mAbs was measured by FACS analysis with FACS Canto II, and the results were analyzed by using FlowJo (version 10.5.0). The EC_{50} was calculated by the non-linear regression of the binding % of serially-diluted mAb with the GraphPad Prism (version 10.0.3).

Expression and purification of S protein

The nucleotide sequence of the full-length Omicron S protein with six proline mutations (F817P, A892P, A899P, A942P, K986P, and V987P) and the furin cleavage site mutation (fm, $^{682}RRAR^{685} \rightarrow {}^{682}GSAG^{685}$) has been codon-optimized for total gene synthesis. The DNA sequence of the S protein was inserted into the mammalian expression vector pcDNA3.1 Myc-His (Invitrogen). This vector includes a foldon trimerization domain derived from phage T4 fibritin, followed by a c-myc epitope and a hexahistidine (His_6) tag as described previously (1, 2). The plasmids were transfected into HEK293 Freestyle cells for protein expression as described previously (1, 2). The recombinant proteins were purified using affinity chromatography by binding to HisPur Cobalt Resin (Thermo Fisher Scientific) overnight in buffer A (50 mM Tris-HCl (pH 7.6), 300 mM NaCl, 5 mM imidazole, and 0.02% NaN_3) at 4 °C. Subsequently, a washing step was performed using buffer B (50 mM Tris-HCl (pH 7.6), 300 mM NaCl, and 10 mM imidazole), followed by elution using buffer C (50 mM Tris-HCl (pH 7.6), 150 mM NaCl, and 150 mM imidazole). The eluted protein was applied onto a size-exclusion chromatography (SEC) column (Superose 6 increase 10/300 GL column; GE Healthcare) in TN buffer (50 mM Tris-HCl (pH 7.6), 150 mM NaCl, and 0.02% NaN_3). The protein concentrations were determined by measuring UV absorbance at 280 nm utilizing a UV–Vis spectrometer (Nano-photometer N60, IMPLEN).

ELISA

Recombinant S protein, S1, S2, RBD or NTD subunit (100 µL) at 1 µg/mL in coating buffer (100 mM $NaHCO_3$ in ddH_2O, pH 8.8) was used to coat onto each well of the 96-well plate (Nunc MaxiSorp ELISA plate, 442404) at 4°C for 16 h. Each well was washed with 200 µl of wash buffer (0.05% Tween20 in PBS), followed by blocking in 100 µl of blocking buffer (2% bovine serum albumin dissolved in PBS) at 37°C for 1 h. After washed for three times, each well was treated with 100 µl of serially-diluted mAb or InVivoMAb human IgG1 isotype control (Bio X Cell, BE0297) in blocking buffer containing 0.05% Tween20, and incubated at 37°C for 1 h. Each well was then washed for three times with wash buffer, followed by incubating with 100 µL of HRP-conjugated anti-human IgG secondary antibody (Jackson ImmunoResearch Inc, 109-035-088) in blocking buffer containing 0.05% Tween20 at 37°C for 1 h. After washed for seven times, each well was added with 100 µl of BD OptEIA™ TMB Substrate Reagent Set (BD Biosciences, 555214). The plate was then kept in dark for 10 min. One hundred µL of 1N sulfuric acid was added to each well to stop the colorimetric reaction. The absorbance was then read at 450 nm on a SpectraMax iD5 plate reader. EC_{50} was calculated by the non-linear regression of the OD_{450} of serially-diluted mAb with the GraphPad Prism (version 10.0.3). The recombinant S proteins and their various domains purchased and used in this study are listed in the table below. All other S proteins were prepared by Dr. Shang-Te Danny Hsu (Academia Sinica, Taiwan).

Manufacturer	Catalog#	Protein
Sino Biological Inc	I-40590-V08B	SARS-CoV-2 (2019-nCoV) Spike S2 ECD-His Recombinant protein
	I-40591-V49H	SARS-CoV-2 (2019-nCoV) Spike S1 NTD-His AVI Recombinant protein
	I-40591-V08H	SARS-CoV-2 (2019-nCoV) Spike S1-His Recombinant protein
	I-40592-V08B-B	SARS-CoV-2 (2019-nCoV) Spike RBD-His Recombinant protein
	I-40589-V08H26	SARS-CoV-2 B.1.1.529 (Omicron) S1+S2 Trimer protein
	I-40591-V08H41	SARS-CoV-2 B.1.1.529 (Omicron) Spike S1 Protein
	I-40591-V08H42	SARS-CoV-2 B.1.1.529 (Omicron) Spike S1 NTD Protein
	I-40589-V08H36	SARS-CoV-2 (BA.2.75) Spike S1+S2 Trimer protein
	I-40589-V08H33	SARS-CoV-2 (BA.5) Spike S1+S2 Trimer protein
	I-40591-V08H46	SARS-CoV-2 (BA.4/BA.5) Spike S1 Protein
	I-40592-V08H130	SARS-CoV-2 (BA.4/BA.5) Spike RBD Protein
	I-40589-V08H45	SARS-CoV-2 XBB.1.5 (Omicron) Spike S1+S2 Trimer Protein
	I-40592-V08H146	SARS-CoV-2 XBB.1.5 (Omicron) Spike RBD Protein
	I-40591-V08H47	SARS-CoV-2 XBB.1.5 (Omicron) Spike S1 Protein
ACRO Biosystems	S2N-C52HF	SARS-CoV-2 BA.1 (Omicron) Spike S2 protein
	SPD-C522J	SARS-CoV-2 BA.1.1 (Omicron) Spike RBD Protein
	SPD-C5246	SARS-CoV-2 (BA.4/BA.5) Spike NTD Protein
	SPN-C524s	SARS-CoV-2 Spike Trimer Protein, His Tag (XBB.1.16/Omicron)

Expression and purification of sfGFP-ACE2 and ACE2-Fc proteins

The sfGFP-ACE2 construct was obtained from Addgene (Plasmid No. 145171), which was deposited by Dr. Erik Procko (University of Illinois Urbana-Champaign, Champaign, USA).

The construct encoding ACE2-Fc protein was a gift from Dr. Han-Chung Wu (Academia Sinica, Taipei, Taiwan). Both secreted recombinant proteins were transiently expressed in Expi293F cell. sfGFP-ACE2 was purified by an anion exchange column (HiTrap Q FF 5 ml; GE Healthcare) eluted by a linear salt gradient from 10 to 1000 mM NaCl, followed by SEC column (Superdex 200 16/600 GL; GE Healthcare) in phosphate buffer. ACE2-Fc protein was purified by Protein A column and followed by SEC column (Superdex 200 16/600 GL; GE Healthcare) in phosphate buffer. The protein concentrations were determined by using the UV absorbance at 280 nm.

Affinity determination of mAbs by biolayer interferometry

The binding kinetics of anti-SARS-CoV-2 mAbs to S protein were measured by BLI with an Octet HTX system (ForteBio). To evaluate the binding kinetics between mAbs and S protein, mAbs were diluted in kinetic buffer (1x PBS, pH 7.4, containing 0.01% BSA, 0.002% Tween 20, and 0.005% NaN_3) to a concentration of 5 µg/ml. Subsequently, the diluted mAbs were applied to Octet ProG biosensors (Sartorius), which has been equilibrated in kinetic buffer, and allowed to interact with the S protein. Binding kinetics were assessed using seven S protein concentrations ranging from 50 nM to 0.782 nM, in 2-fold dilution. The assay mainly comprised the following steps: (1) baseline: 60 sec with kinetic buffer; (2) loading: 120 sec with mAbs; (3) baseline: 60 sec with kinetic buffer; (4) association: 300 sec with S protein; and (5) dissociation: 300 sec with kinetic buffer. K_D values were calculated by using a 1:1 global fit model (Octet).

To evaluate the effect of O5C2 on the inhibition of the binding of S variants to ACE2, a stock solution of sfGFP-ACE2 was diluted to at 10 µg ml^{-1} for immobilization onto High Precision Streptavidin (SAX) biosensors (Sartorius) in assay buffer (PBS, 0.02% tween-20, 0.1% BSA) according to manufacturer's instructions. Stock solutions of S proteins from Omicron variants were serially diluted to 50, 25, 12.5, 6.25, 3.125 and 1.5625 nM in assay buffer for independent binding using an OctetRED 96 biolayer interferometer (ForteBio). The association and dissociation were monitored over 600 sec and 900 sec, respectively. The six independent BLI sensorgrams with different S protein concentrations were baseline corrected using double reference, that is, biosensors without sfGFP-ACE2 were used to collect sensorgrams with another set of S protein samples to minimize nonspecific binding and to extract the actual responses from the protein-protein interactions. The double reference subtracted data were fitted to a 1:1 binding model using Data Analysis v.10.0 software (ForteBio). For S protein-O5C2 neutralization analysis, O5C2 were individually mixed with an equal molar ratio of S proteins at a final concentration of 25 nM, and incubated at room temperature for 30 mins before BLI measurements using the same sfGFP-ACE2-immobilized biosensors with 400 sec of association and 150 sec of dissociation. The results were exported and replotted Prism v.9 (GraphPad).

Plaque reduction neutralization (PRNT) test

PRNT experiments were done in the BSL-3 facility in the Institute of Biomedical Sciences, Academia Sinica, Taiwan. SARS-CoV-2 variants of WT SARS-CoV-2 (hCoV-19/Taiwan/4/2020, GISAID accession ID: EPI_ISL_411927), Delta SARS-CoV-2 hCoV-19/Taiwan/1144/2021, GISAID accession ID: EPI_ISL_5854263, BA.5 (hCoV-19/Taiwan/689423/2022, GISAID accession ID: EPI_ISL_17696988) and XBB.1 SARS-CoV-2 (hCoV-19/Taiwan/984581/2022, GISAID accession ID: EPI_ISL_17068748) were used in the PRNT. Serially-diluted O5C2 mAb, starting from 10 µg/ml, in PBS was incubated with live virus of 100 plaque-forming units (PFU) at 37°C for 1 hour. The pre-seeded Vero-

E6 cells were then added with the mAb and SARS-CoV-2 virus mixtures and incubated at 37°C for 1 hour. The culture medium containing virus was replaced with DMEM containing 1% methyl-cellulose and 2% FBS. The cells were further cultured for additional 4 days, followed by fixation in 10% formaldehyde and staining in 0.5% crystal violet for 20 min. The plaque area of each well was calculated with Image J. The inhibition % by mAb is defined as the percentage of decrease in plaque area as compared to the wells without mAb (virus + cells). The half maximal concentration of $PRNT_{50}$ was calculated by the non-linear regression of inhibition % of the serially-diluted O5C2 mAb by GraphPad Prism (version 10.0.3).

Preparation of NK-92MI cell line expressing FcγRIIIA-FcRγ fusion protein
NK-92 MI cell line was acquired from the Bioresource Collection and Research Center (Hsinchu, Taiwan). Because NK-92 cell line does not express CD16 (FcγRIII) (3), we engineered NK92 MI cells that express FcγRIIIA-FcRγ for ADCC assay. The construct for the FcγRIIIA-FcRγ fusion protein, was similar to the one described by others (4). Briefly, the cDNA for human FcγRIIIA (gene symbol *FCGR3A*; V176 (aka V158) variant, NM_000569.8:c.526T>G) was obtained by PCR from a Human Bone Marrow Marathon®-ready cDNA Library (Clontech/Takara), and the segment encoding the extracellular domain (amino acid #1–206) was amplified by PCR. The cDNA for human FcRγ (gene symbol: *FCER1G*) was cloned as described (5), and the segment encoding the transmembrane–intracellular domain (amino acid #24–87) was amplified by PCR, subsequently cloning into pcDNA3.1(−) vector and yielding the plasmid FcγRIIIA-FcRγ/pcDNA3.1. The cDNA for FcγRIIIA-FcRγ fusion protein was subcloned to pLAS5w.pPuro transfer vector (RNA Technology Platform and Gene Manipulation Core, Academia Sinica) and packaged into a lentivirus by transient co-transfection of 293T cells with packaging constructs (pMD.G and pCMVDR8.91). The supernatant was collected, cleared by centrifugation (1,100 × g for 10 min), and used for the transduction of NK-92 MI cells by spinoculation (1,000 × g for 30 min) in the presence of 10 μg/mL polybrene. Transduced cells were selected for 7 days in the presence of 1 μg/mL puromycin in the complete medium. The cells that express CD16 (FcγRIII), NK-92 MI FcR, were stained with APC anti-human CD16 Antibody (BioLegend, 302012) and sorted at the Flow Cytometry Core Facility, the Institute of Biomedical Sciences, Academia Sinica. and expanded. Detailed cloning procedures and primer information will be available upon request.

Competitive ELISA
mAbs were conjugated with HRP using HRP Conjugation Kit (Abcam, Cat. No. ab102890). The reaction was operated according to the manufacturer's instruction with molar ratio IgG:HRP=1:4. HRP conjugated mAbs were used as competitive antibodies. Each well of F96 Maxisorb Nunc-Immuno plate (Thermo Fisher Scientific, Cat. No. 442404) was coated with 0.2 μg of SARS-CoV-2 (BA.5) S S1+S2 trimer protein (Sino Biological Inc., Cat. No. 40589-V08H33) diluted in 100 mM $NaHCO_3$ (pH8.8) at 4 °C overnight. After plates were blocked with 2% BSA in PBS for 1 h at 37 °C, 50 μl of serially diluted unlabeled test mAbs were incubated with BA.4/5 protein-precoated ELISA plates followed by adding 50 μl of competitive antibody O4C6-HRP (250 ng/mL), O5C2-HRP (100 ng/mL), O5C6-HRP (62.5 ng/mL), O5F4-HRP (62.5 ng/mL) or O5G7-HRP (1000 ng/mL), respectively. After incubating at 37 °C for 1 h, the plates were washed with PBST. The color was developed by treating with 100 μl of BD OptEIA™ TMB subtract reagent (BD Biosciences, Cat. No. 555214) for 10

min and then stopped by adding 100 µl of 1M H_2SO_4. The absorbance at 450 nm was measured by SpectraMax iD5 (Molecular Devices).

High-throughput transcriptome dataset processing

Standard low-quality read filtering and quality checks for bulk RNA-seq dataset were conducted using the program fastp (6), with the default parameters (quality score > 20, minimum length = 15 bp). The filtered reads are mapped to the latest *Mus musculus* reference genome, GRCm39, using the program STAR aligner with default parameters (7). The genome and gene model references were retrieved from the NCBI (ncbi.nlm.nih.gov/datasets/genome/GCF_000001635.27/). The mapping metrics were examined using Qualimap to quantify the mapping rate of exonic, intronic, and intergenic regions (8). The program featureCounts from Subread packages were used to count the mapped reads for each gene (9), and the gene model GTF file of GRCm39 (ncbi.nlm.nih.gov/datasets/gene/GCF_000001635.27/) was used as the gene model reference.

Differential gene expression analysis and data visualization

The mapped read counts data were imported into R environment (10) and analyzed using DESeq2 packages (11). The differentially expressed genes are selected based on the $\log_2$ fold change and *p*-value cut-off values of 1.0 and 0.05, respectively. The GO enrichment analysis of biological process domains were performed using goseq packages (12) using these selected differentially expressed genes. All the figures are plotted using the ggplot2 packages in R language (13), and the EnchancedVolcano packages was specifically used to generate the volcano plot (14). All the codes for the RNA-seq analysis and visualization are provided in the Supplementary Table 4.

Supplemental References

1. Yu PY, et al. Biophysical and structural characterizations of the effects of mutations on the structure-activity relationships of SARS-CoV-2 spike protein. *Methods Enzymol.* 2022;675:299-321.
2. Yang TJ, et al. Effect of SARS-CoV-2 B.1.1.7 mutations on spike protein structure and function. *Nat Struct Mol Biol.* 2021;28(9):731-739.
3. Gong JH, et al. Characterization of a human cell line (NK-92) with phenotypical and functional characteristics of activated natural killer cells. *Leukemia.* 1994;8(4):652-658.
4. Clemenceau B, et al. Antibody-dependent cellular cytotoxicity (ADCC) is mediated by genetically modified antigen-specific human T lymphocytes. *Blood.* 2006;107(12):4669-4677.
5. Angata T, et al. Siglec-15: an immune system Siglec conserved throughout vertebrate evolution. *Glycobiology.* 2007;17(8):838-846.
6. Chen S. Ultrafast one-pass FASTQ data preprocessing, quality control, and deduplication using fastp. *iMeta.* 2023;2(2):e107.
7. Dobin A, et al. STAR: ultrafast universal RNA-seq aligner. *Bioinformatics.* 2013;29(1):15-21.
8. Okonechnikov K, et al. Qualimap 2: advanced multi-sample quality control for high-throughput sequencing data. *Bioinformatics.* 2016;32(2):292-294.
9. Liao Y, et al. featureCounts: an efficient general purpose program for assigning sequence reads to genomic features. *Bioinformatics.* 2014;30(7):923-930.
10. R Core Team. *R: A Language and Environment for Statistical Computing.* R Foundation for Statistical Computing; 2020.
11. Love MI, et al. Moderated estimation of fold change and dispersion for RNA-seq data with DESeq2. *Genome Biol.* 2014;15(12):550.
12. Young MD, et al. Gene ontology analysis for RNA-seq: accounting for selection bias. *Genome Biol.* 2010;11(2):R14.
13. Wickham H. *ggplot2: Elegant Graphics for Data Analysis.* Springer-Verlag New York; 2016.
14. Blighe K, Rana, S., & Lewis, M. *EnhancedVolcano: Publication-ready volcano plots with enhanced colouring and labeling.* 2020.

Supplemental Table 1, 4, 6 and 7 are in separate files.

Supplemental Table 2. Cryo-EM data collection, refinement and validation statistics

	S-BQ.1:ACE2 (EMD-38201, PDB 8XAL)	S-BQ.1:O5C2 (EMD-38216, PDB 8XBF)
**Data collection and processing**		
Microscope	Titan Krios	Titan Krios
Detector	K3	K3
Magnification	x 81000	x 81000
Voltage (kV)	300	300
Electron exposure (e–/Å^2)	50	50
Defocus range (μm)	0.8-1.8	0.8-1.8
Pixel size (Å)	1.1	1.1
Symmetry imposed	C1	C1
Initial particle images (no.)	2,275,984	2,071,251
Final particle images (no.)	128,951	1,255,741
Map resolution (Å)	3.2	3.6
FSC threshold	0.143	0.143
**Refinement**		
Initial model used (PDB code)	8DM5	S-BQ.1:ACE2
Model resolution (Å)	2.6	2.3
FSC threshold	0.143	0.143
Model composition		
Non-hydrogen atoms	36,628	30,104
Protein residues	4,579	3,833
Ligands	NAG: 33	NAG: 18
B factors (Å^2)		
Protein	92.97	120.00
Ligand	105.46	112.39
R.m.s. deviations		
Bond lengths (Å)	0.003	0.004
Bond angles (°)	0.605	0.617
Validation		
MolProbity score	2.06	2.68
Clashscore	9.11	16.44
Rotamer outliers (%)	1.65	5.28
Ramachandran plot		
Favored (%)	93.89	93.72
Allowed (%)	6.08	6.28
Disallowed (%)	0.02	0.00

Supplemental Table 3. Mutations in the RBD of Omicron S proteins

Res. No.	Wuhan	BA.1	BA.2	BQ.1
339	G	D	D	D
371	S	L	F	F
373	S	P	P	P
375	S	F	F	F
376	T	T	A	A
405	D	D	N	N
408	R	R	S	S
417	K	N	N	N
440	N	K	K	K
444	K	K	K	T
446	G	S	G	G
452	L	L	L	R
460	N	N	N	K
477	S	N	N	N
478	T	K	K	K
484	E	A	A	A
486	F	F	F	V
493	Q	R	R	Q
496	G	S	G	G
498	Q	R	R	R
501	N	Y	Y	Y
505	Y	H	H	H

Res. No. is an abbreviation for residue number.

Supplemental Table 5. The high-throughput sequencing and mapping matrices of the four BA.5 infected mice that were pre-treated with PBS or O5C2.

The raw reads of each sample are first filtered with *fastp* to obtain high quality (> Q30) filtered reads, and mapped to the latest *Mus musculus GRC mouse 39* (mm39) reference genome using *STAR aligner*. The uniquely mapped reads are the reads that are unambiguously mapped (only mapped once) to the target reference sites. The read counts of each gene are counted using *featureCounts* for the downstream differential gene expression (DGE) analysis.

	PBS-1	PBS-2	O5C2-1	O5C2-2
**Number of reads**	93,271,510	91,337,486	81,100,157	89,638,549
**Filtered read**	92,411,690 (99.08%)	90,550,062 (99.14%)	80,400,617 (99.14%)	88,859,178 (99.13%)
**Q30 Bases**	95.16%	95.30%	95.13%	95.18%
**Reads mapped to genome**	89,067,572 (96.38%)	84,833,195 (93.39%)	76,558,647 (95.22%)	85,385,058 (96.09%)
**Uniquely mapped:**				
**Genome**	84,877,973 (95.30%)	79,081,209 (93.22%)	71,938,236 (93.96%)	79,628,917 (93.36%)
**Intergenic regions**	3,459,918 (4.11%)	3,498,542 (4.46%)	3,076,388 (4.32%)	5,219,421 (6.61%)
**Intronic regions**	52,475,990 (62.27%)	46,435,923 (59.18%)	41,573,852 (58.38%)	45,100,210 (57.11%)
**Exonic regions**	28,340,338 (33.63%)	28,533,622 (36.36%)	26,559,020 (37.3%)	28,658,049 (36.29%)
**rRNA (mito + nuc)**	27,041 (< 0.01 %)	36,825 (< 0.01 %)	30,212 (< 0.01 %)	10,123 (< 0.01 %)
**Reference genome**	*Mus musculus* GRC Mouse build 39 (GCF000001365.27)			
**Gene model reference**	GRCm39genome.gtf			
**Mapping tools**	STAR v2.7.10b			
**Filtering tools**	fastp v0.22.0			
**Counting tool**	featureCounts v2.0.6			

Supplemental Figures and Legends

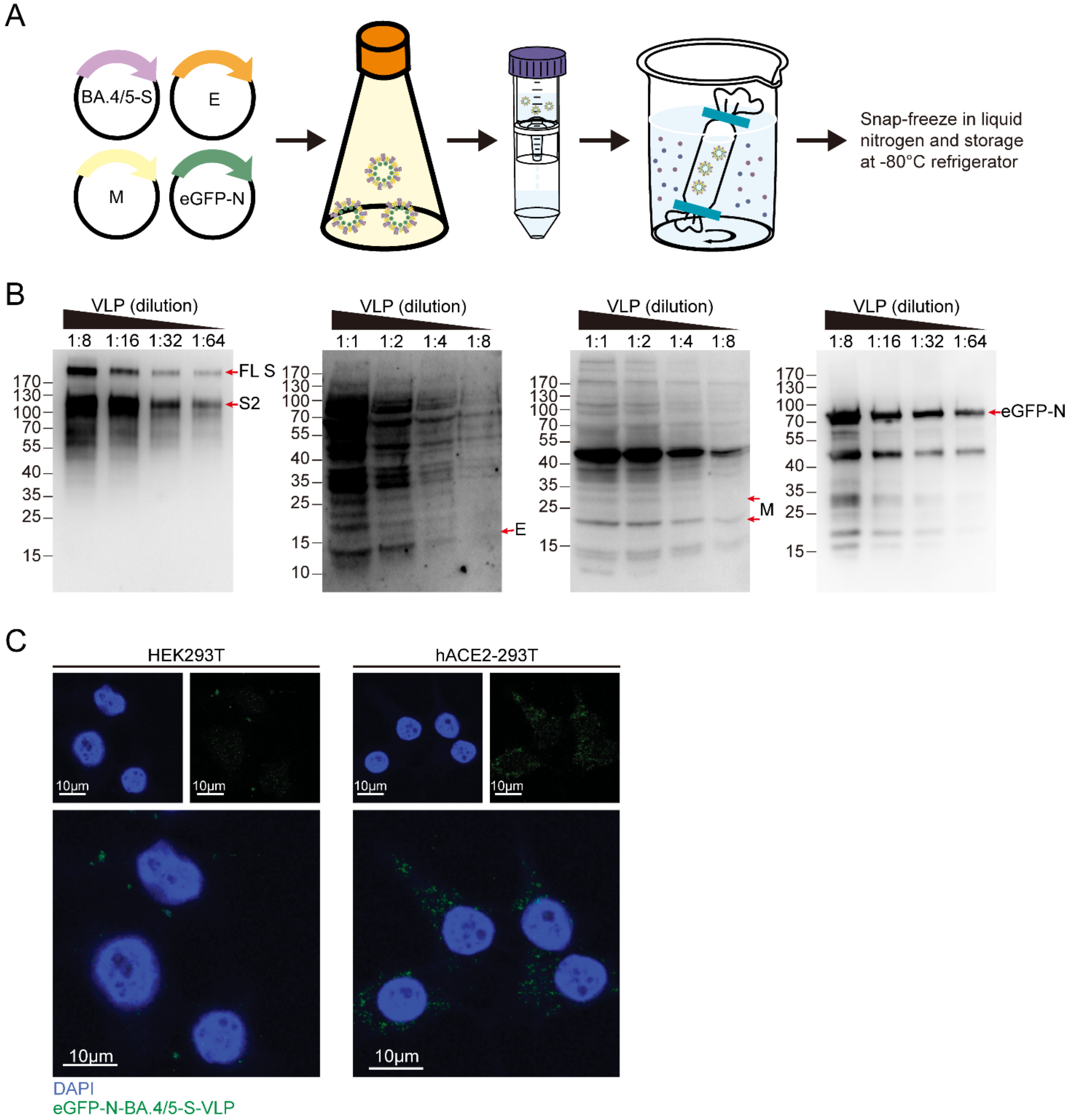

Supplemental Figure 1. Generation of BA.4/5 S protein expressing VLP

(A) The schema illustrates the flowchart of production of EGFP-N-BA.4/5-S-VLP. **(B)** Immunoblotting showing the expression of S, E, M and EGFP-N proteins (from left to right as indicated) by EGFP-N-BA.4/5-S-VLP. Serially diluted VLP, as indicated, were used. **(C)** Confocal microscopy analysis showing the binding of EGFP-N-BA.4/5-S-VLP to HEK293T or hACE2-293T cells. FL S, full-length S protein. Scale bar is indicated.

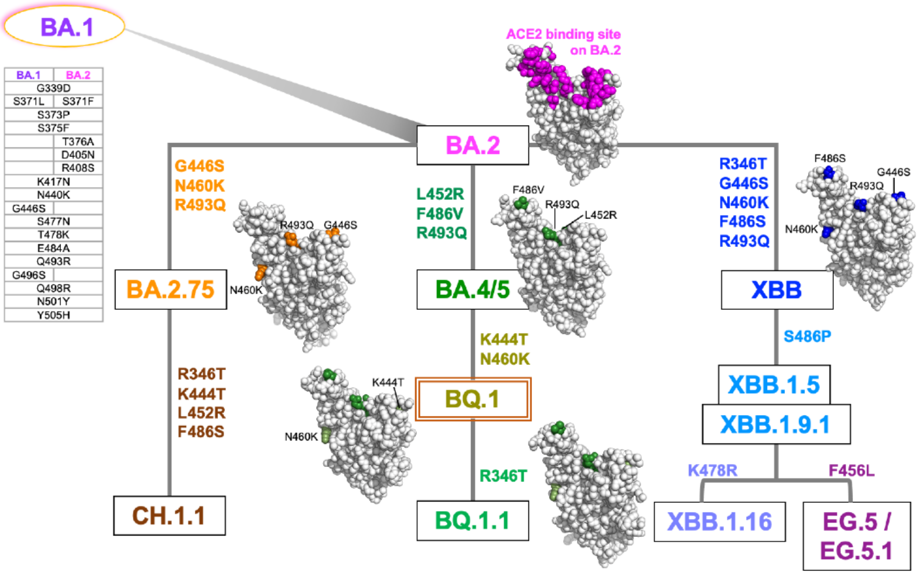

Supplemental Figure 2. A schematic of SARS-CoV-2 Omicron subvariant evolution, indicating the mutations on the RBD

Mutation sites on the RBD of each Omicron sublineage are indicated. BA.2 has six unique and 12 shared mutations compared with BA.1. The magenta residues on BA.2 are the ACE2 interacting residues. The additional mutation sites compared with BA.2 are colored in orange (BA.2.75), brown (CH.1.1), dark green (BA.4/5), light green (BQ.1 and BQ.1.1), blue (XBB series), light blue (XBB.1.5 and XBB.1.9.1), light purple (XBB.1.16) and violet (EG.5 and EG.5.1).

A

Donor	Vaccination	Days after quarantine
O4	M+M+BNT	13
O5	M+M+M	12
O9	AZ+AZ+M	6

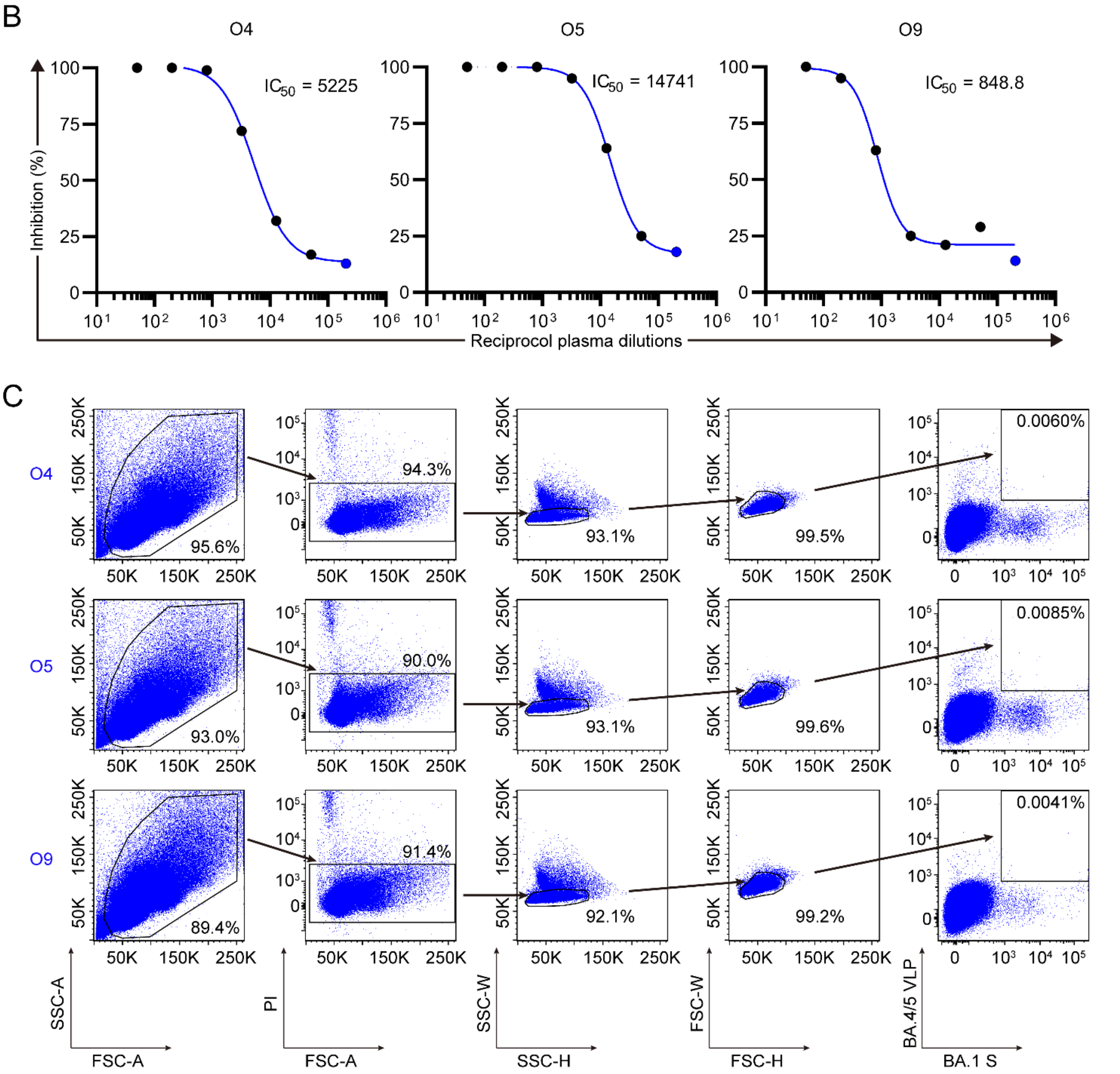

Supplemental Figure 3. Vaccination history of three donors, the neutralizing activity of their plasma against BA.4/5-pseudotyped virus, and the percentage of peripheral blood B cells targeting both BA.1 S protein and EGFP-N-BA.4/5-S-VLP
(A) The order and type of vaccines received (M: Moderna, BNT: Pfizer-BioNTech, AZ: AstraZeneca) before breakthrough infection, as well as the days on which blood samples were collected from the three donors after testing negative by a rapid test. **(B)** BA.4/5 pseudotyped virus neutralization potency of heat-inactivated plasma from the three donors.

IC50 (dilution factor) is indicated. **(C)** Peripheral blood B cells purified by α-human CD19 magnetic beads were stained with PI, BA.1 S protein and EGFP-N-BA.4/5-S-VLP, followed by single live cell sorting. The gating strategy and frequency of single live B cells binding to both BA.1 S protein and EGFP-N-BA.4/5-S-VLP is shown. The IC_{50} values in **B** were calculated by fitting a curve using a four-parameter dose-response nonlinear regression analysis.

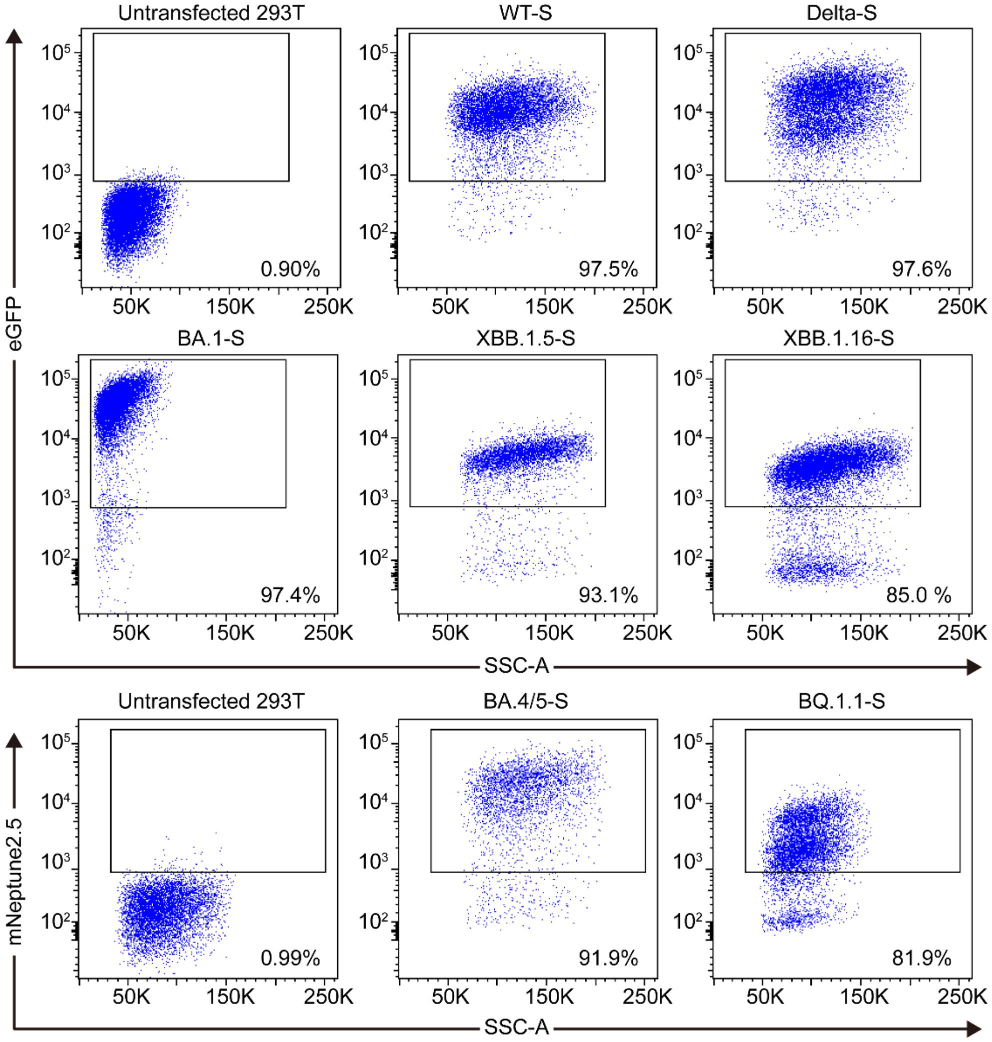

Supplemental Figure 4. Generation of S-293T cells expressing S proteins from various SARS-CoV-2 variants

HEK293T cells were transfected with vectors expressing P2A-EGFP or P2A-mNeptune2.5-tagged S protein of various indicated SARS-CoV-2 variants. GFP^+ or mNeptune2.5^+ cells were selected by a cell sorter 14 days after transfection. The sorted cells were maintained in culture, followed by individually sorting cells expressing high-intensity fluorescence. The isolated single cell clones were expanded and further sorted one to three times until the GFP^+ or mNeptune2.5^+ rate stably reached > 80%, as shown.

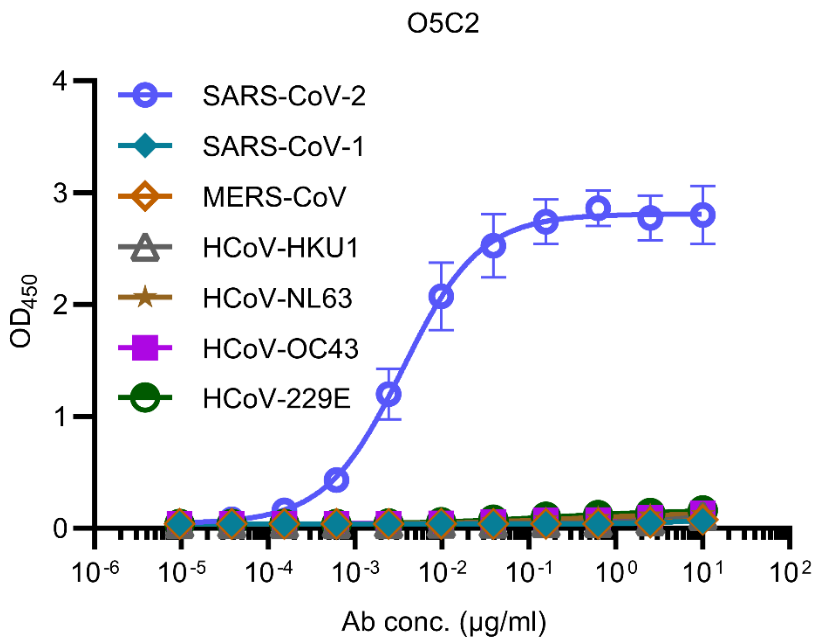

Supplemental Figure 5. O5C2 mAb does not recognize S proteins of non-SARS-CoV-2 human coronaviruses

ELISA showing O5C2 binds to the S ectodomain of WT SARS-CoV-2 but not SARS-CoV-1, MERS-CoV, HCoV-HKU1, HCoV-NL63, HCoV-OC43 or HCoV-229E. The results for SARS-CoV-2 are three replicates from two independent experiments and are presented as the mean ± s.e.m. Data for SARS-CoV-1, MERS-CoV and HCoVs are single results from one experiment. The fit-curves were generated by nonlinear regression.

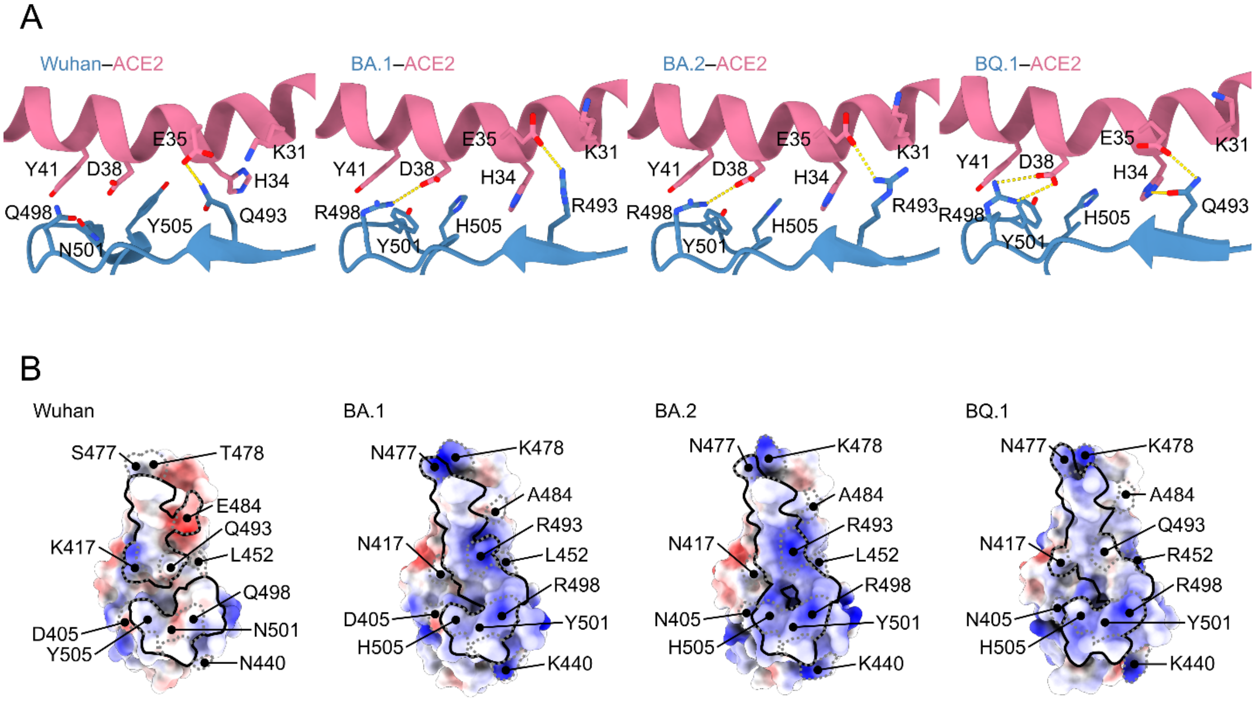

Supplemental Figure 6. Molecular basis of ACE2-binding by the RBD of S variants
a. Expanded views of the RBD:ACE2 binding interface with the interacting residues shown in stick representations. The oxygen and nitrogen atoms are colored red and blue, respectively. The color scheme is the same as for Figure 3b. **b.** Electrostatic potential surfaces around the receptor binding motifs (RBMs). The RBDs are shown in surface representations and color-ramped from red to white to blue, corresponding to negatively, neutrally and positively charged surfaces. Black solid lines outline the RBMs, defined by the residues that showed intermolecular contacts with ACE2 within 5 Å. Gray dotted lines outline the locations of the mutated residues specific to the particular variant. The structures of ACE2 in complex with the RBD of the Wuhan, BA.1 and BA.2 strains were taken from Protein Data Bank (PDB) under the entries: 6M0J, 7T9L and 8DM6, respectively.

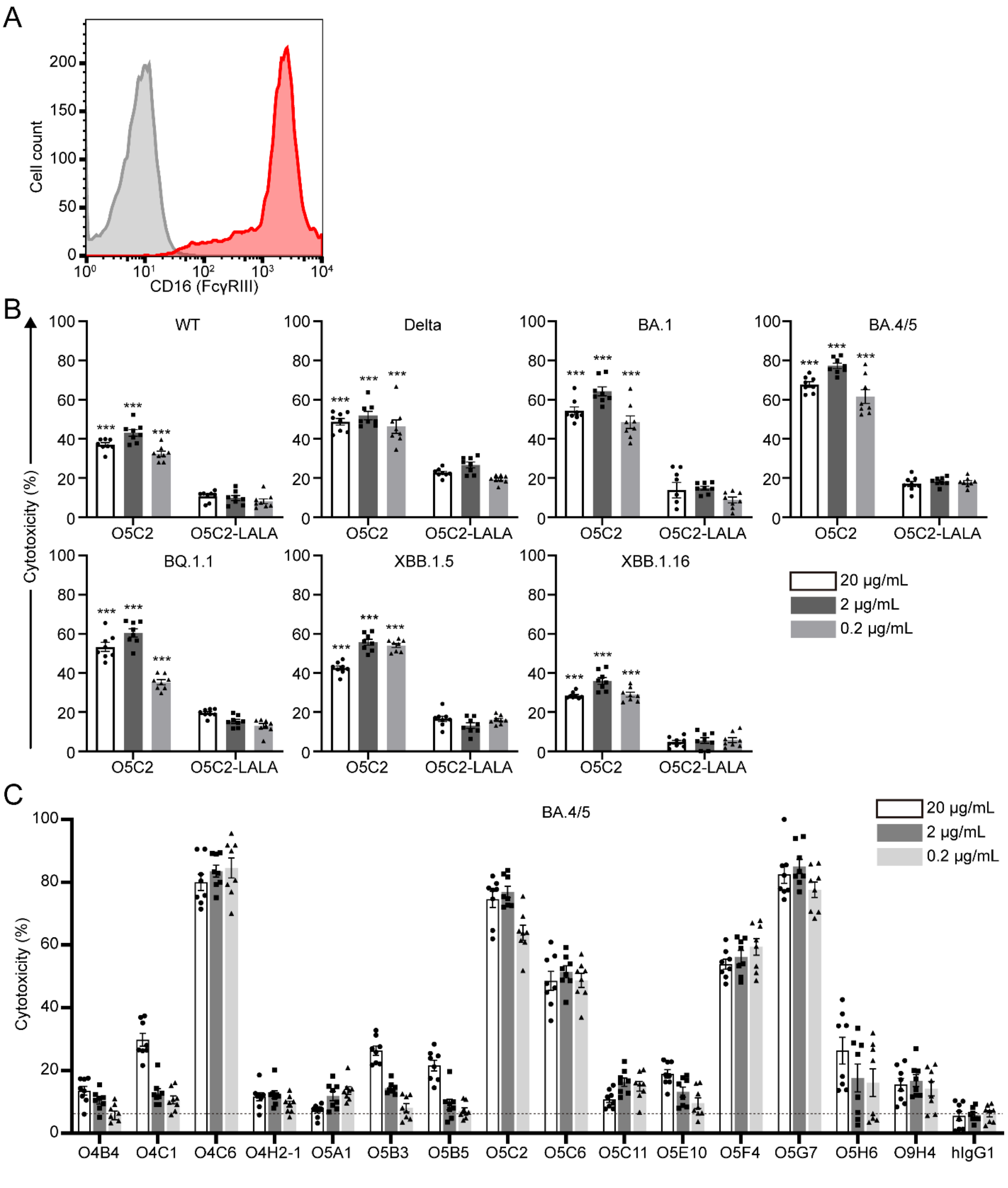

Supplemental Figure 7. ADCC killing assays reveal that O5C2 has a broad spectrum of ADCC activity and that five mAbs in this study possess ADCC

(A) Expression of FcγRIIIA-FcRγ fusion protein on engineered NK-92 MI-FcR cells. The cDNA for FcγRIIIA-FcRγ fusion protein was introduced into NK-92 MI cells by lentiviral transduction, and the drug-selected cells were subjected to cell sorting to enrich the cells expressing CD16 (i.e., FcγRIII). The sorted cells were expanded and probed with anti-

CD16 (red histogram) or the isotype control (gray histogram) to confirm the stable expression of the recombinant fusion protein by FACS analysis. **(B)** ADCC activity of O5C2 and O5C2-LALA at various doses (20, 2 and 0.2 μg/mL) to mediate the killing of the indicated S-293T cells by NK-92 MI-FcR cells. **(C)** ADCC activity of the 15 identified mAbs at various doses (20, 2 and 0.2 μg/mL) to mediate the killing of BA.4/5 S-293T cells by NK-92 MI-FcR cells. Human IgG (hIgG) was used as the negative control. Results in **B** and **C** represent the mean ± s.e.m., and are from one experiment with two independent biological replicates (n=8). Data in **B** were analyzed by the two-tailed Student's *t*-test to compare the killing activity of O5C2 and O5C2-LALA at the same concentration. Statistical analysis in **B** was conducted by comparing the killing activity of O5C2 and O5C2-LALA at the same concentration (***$p<0.001$).

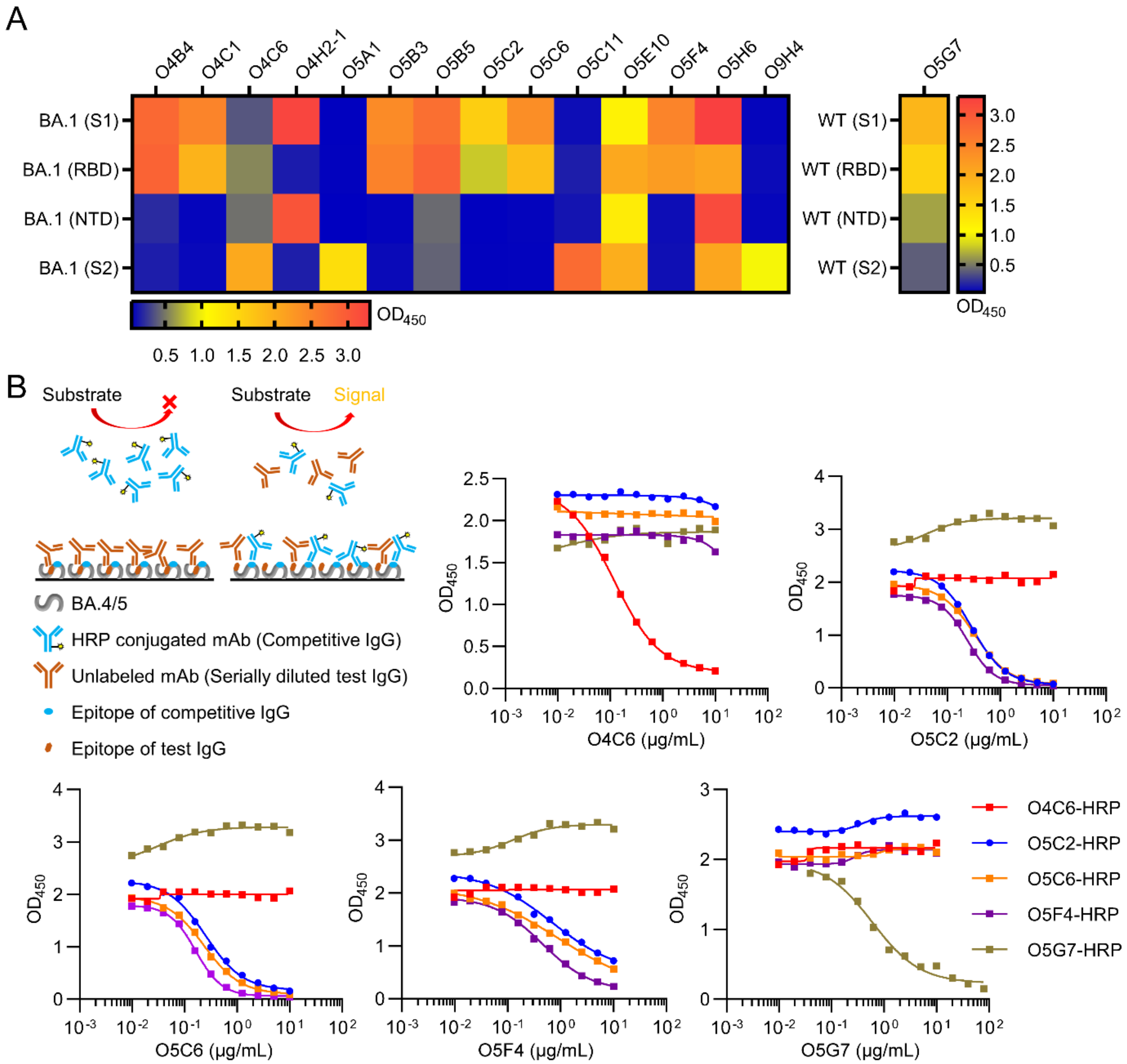

Supplemental Figure 8. Identification of the domains of S proteins targeted by 15 mAbs by ELISA and competitive ELISA

(A) ELISA showing O5G7 and the other 14 identified S protein reactive mAbs binding to different domains, including S1, RBD, NTD and S2 of the WT (O5G7) or BA.1 (others) S proteins, respectively. **(B)** Competitive ELISA using the five identified mAbs with ADCC activity—O4C6, O5C2, O5C6, O5F4 and O5G7—conjugated to HRP prior to the assay. The 96-well plates were coated with BA.4/5 full-length S protein (200 ng/well), with serially diluted unconjugated mAbs added, along with the indicated HRP-conjugated mAbs. Absorbance was detected at 450 nm.

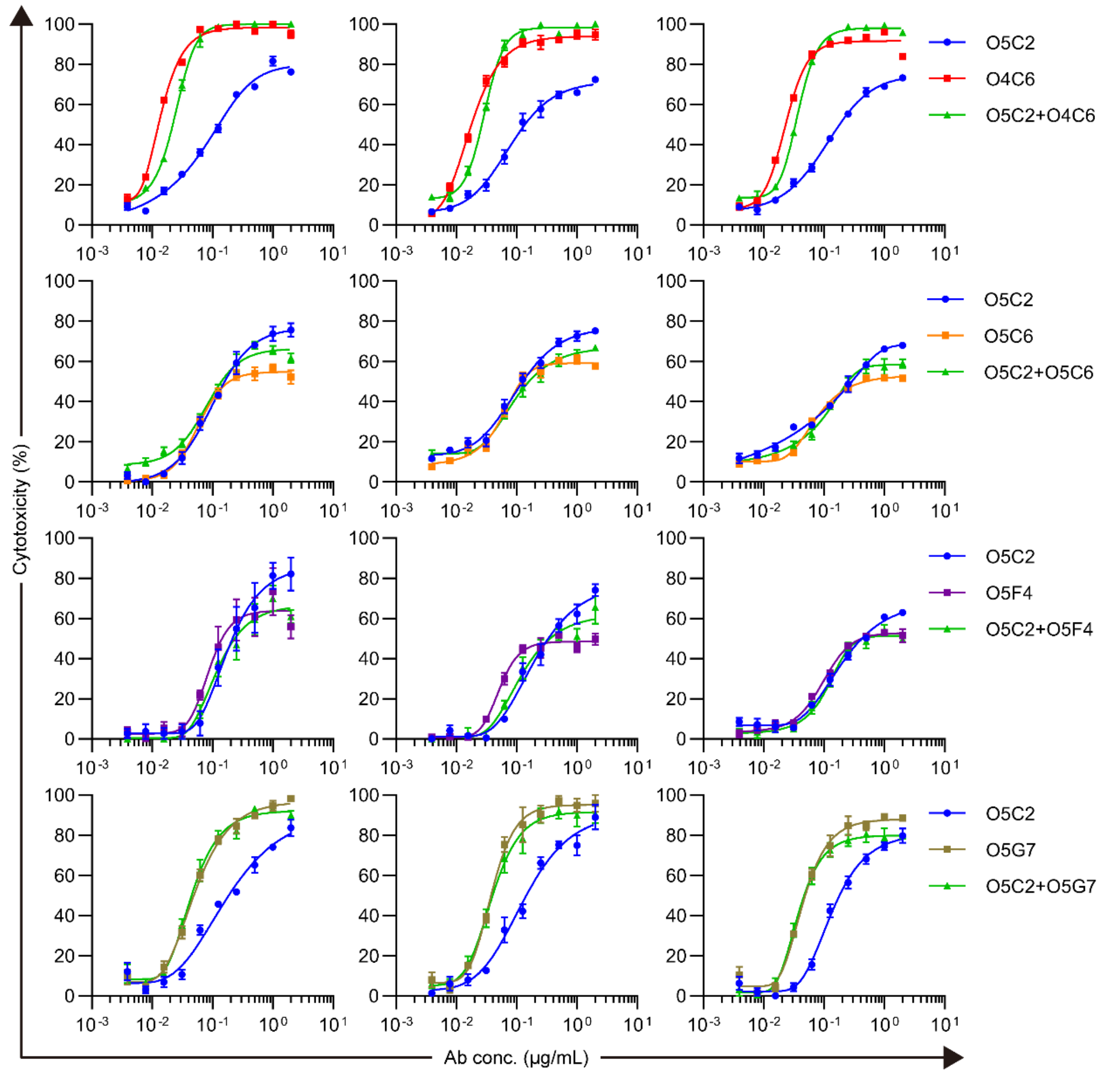

Supplemental Figure 9. O5C2 works in concert with other mAbs in ADCC
ADCC activity of O5C2 and the other individual mAbs, including O4C6, O5C6, O5F4 and O5G7, and the combination of O5C2 with O4C6, O5C6, O5F4 or O5G7 (1:1 ratio) to kill BA.4/5 S-293T cells mediated by NK-92 MI-FcR cells. Three independent experiments are shown (n=4 in each independent experiment).

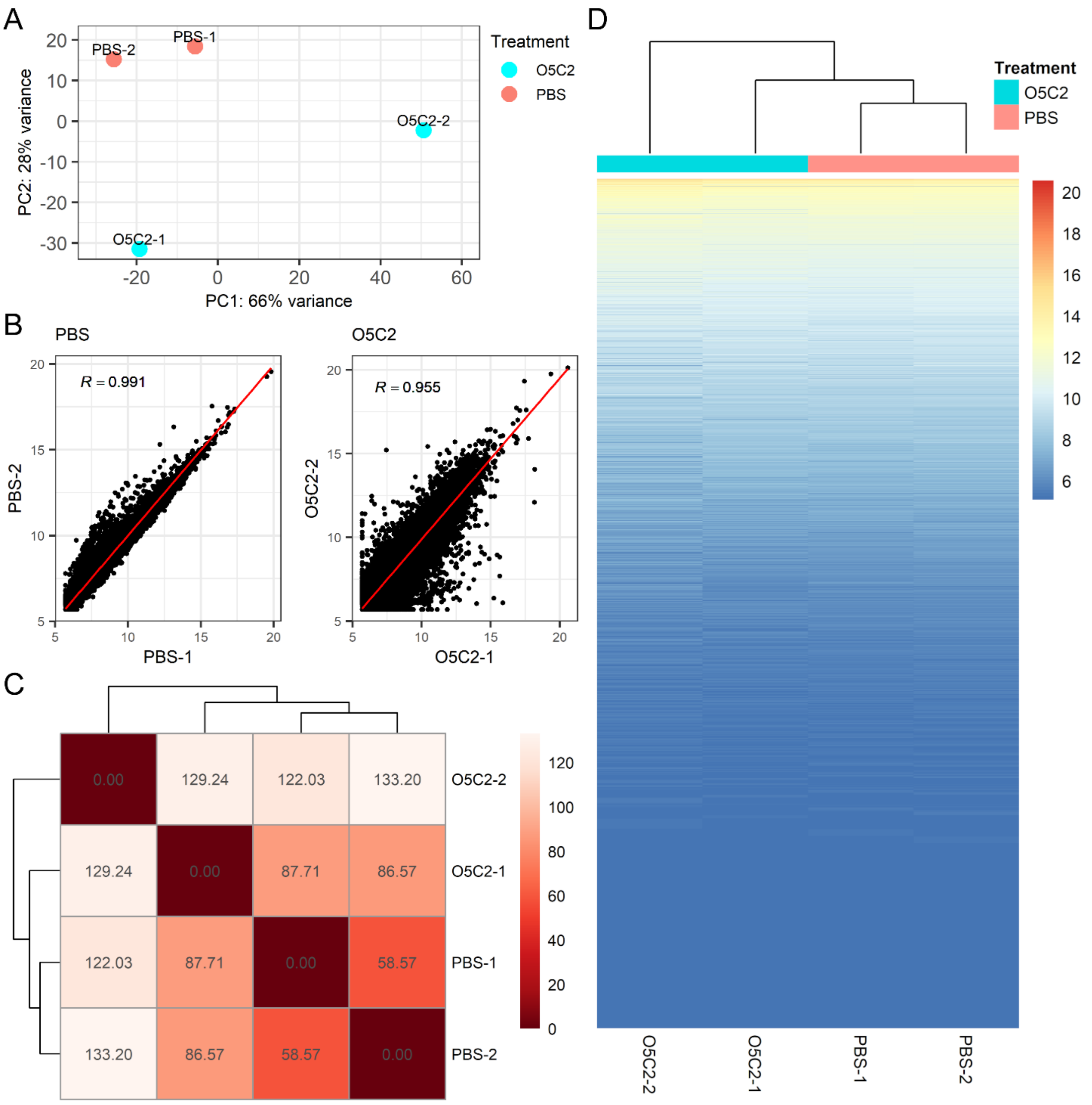

Supplemental Figure 10. Distinct gene expression cluster in infected mice treated with PBS or O5C2

(A) PCA plot shows the clustering of samples treated with O5C2 antibody (light blue), and PBS (red) as the control. **(B)** Dotplots show the correlation of normalized gene expression between biological replicates with the same treatment. The value on the top left of each plot indicates Pearson's correlation coefficient (R). **(C)** Plot shows the distance matrix between samples. The color gradient indicates similarity: as the red color becomes darker, the PBS and O5C2 treated samples become closer and more similar. **(D)** Heatmap analysis showing the expression level of each gene for the samples. Each row indicates

a single gene. Samples treated with PBS clustered together based on their gene expression profile.
